# Structure of the Lipopolysaccharide from *Paenalcaligenes hominis*: A Chemical Perspective on Immune Recognition

**DOI:** 10.1021/jacsau.5c00441

**Published:** 2025-06-24

**Authors:** Ferran Nieto-Fabregat, Marcello Mercogliano, Alessandro Cangiano, Giuseppe Vitiello, Emanuela Andretta, Luke A. Clifton, Adele Vanacore, Lorena Buono, María Asunción Campanero-Rhodes, Dolores Solís, Cristina Di Carluccio, Giovanni Pecoraro, Antonio Molinaro, Giovanni Smaldone, Jeon-Kyung Kim, Dong-Hyun Kim, Luigi Paduano, Flaviana Di Lorenzo, Alba Silipo

**Affiliations:** † Department of Chemical Sciences and Task Force for Microbiome Studies, 9307 University of Naples Federico II, Via Cinthia 4, 80126 Naples, Italy; ‡ CSGI, Center for Colloid and Surface Science, Sesto Fiorentino, Florence 50019, Italy; § Department of Chemical, Materials and Production Engineering, University of Naples Federico II, P. le Tecchio 80, 80125 Naples, Italy; ∥ ISIS Pulsed Neutron and Muon Source, Science and Technology Facilities Council, 97008 Rutherford Appleton Laboratory, Harwell Science and Innovation Campus, Didcot, Oxfordshire OX11 OQX, U.K.; ⊥ 591458 IRCCS SYNLAB SDN, Via G. Ferraris 144, 80146 Naples, Italy; # Instituto de Química Física Blas Cabrera, CSIC, Serrano 119, 28006 Madrid, Spain; ¶ CIBER de Enfermedades Respiratorias (CIBERES), Avda Monforte de Lemos 3-5, 28029 Madrid, Spain; ∇ CEINGE Biotecnologie Avanzate Franco Salvatore, Via Gaetano Salvatore, 486, 80131 Naples, Italy; ○ Neurobiota Research Center, College of Pharmacy, 26723 Kyung Hee University, Seoul 02447, Korea

**Keywords:** Paenalcaligenes hominis, lipopolysaccharide, innate immunity, NMR spectroscopy, mass spectrometry

## Abstract

Gram-negative bacterium *Paenalcaligenes hominis*, which is increasingly prevalent in elderly individuals, is associated with cognitive decline and gut–brain axis dysfunction. Here, we present a comprehensive structural characterization of *P. hominis* lipopolysaccharide (LPS), a key modulator of immune recognition and the main component of its outer membrane. Using a multidisciplinary approach combining chemical, spectroscopic, spectrometric, biophysical and computational methods, we unveil a unique O-antigen characterized by a trisaccharide repeating unit containing rhamnose and glucosamine, displaying nonstoichiometric O-acetylation and a terminal methylated rhamnose capping the saccharide chain. Furthermore, we disclose a short core oligosaccharide and a Lipid A composed of penta- to tetra-acylated species. Notably, this LPS exhibits reduced activation of Toll-Like Receptor-dependent signaling compared to the highly immunostimulatory *Escherichia coli* LPS and elicits a poor pro-inflammatory cytokine response. Moreover, *P. hominis* LPS exhibits selective binding to immune lectins such as Ficolin-3 and Galectin-4, as shown by the microarray assays. This raises the possibility that lectin-mediated recognition may represent an alternative route of immune engagement, which could help explain altered immune responses observed in elderly individuals. These findings provide a molecular basis for further exploring the role of *P. hominis* LPS in microbiota-induced immune modulation and its possible impact on age-related inflammatory and neurodegenerative conditions.

## Graphical abstract

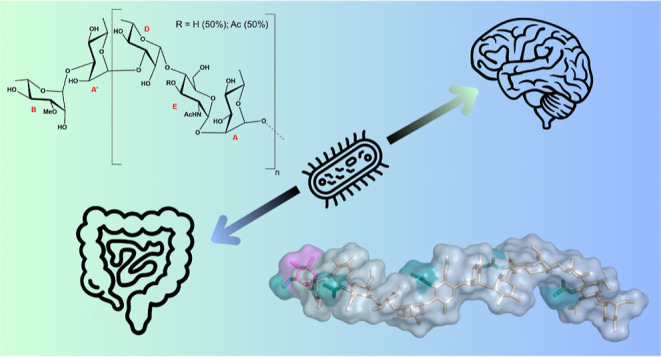

## Introduction

The human microbiome, a varied community of microbes, genes and their products, colonizes all parts of the body.^1^ The gut microbiota, specifically, plays a critical role in human health and disease,^2,3^ interacting closely with the intestinal immune system to maintain immune balance. This crosstalk not only supports immune homeostasis but also enables the body to recognize and combat pathogens, thereby preventing dangerous infections while promoting tolerance toward beneficial microbes.^4,5^ However, despite the number of studies still ongoing in this field, the mechanisms of host–microbe interactions remain a mystery to be solved. Even more intriguing is the so-called “gut–brain axis” (GBA), a bidirectional communication network linking the enteric and central nervous systems.^6^ The GBA encompasses anatomical, endocrine, humoral, metabolic and immunological pathways and plays a key role in both gastrointestinal health and psychological well-being.^7^ It integrates intestinal functions, connecting the brain’s emotional and cognitive centers with peripheral gut mechanisms like immune activation, intestinal permeability, enteric reflexes, and entero-endocrine signaling.^8^ Disruptions in this axis, whether from endogenous or exogenous factors, such as aging, have been linked to the acceleration of psychiatric disorders including depression and anxiety,^9^ and also to conditions like autism, Alzheimer’s disease,^10^ and inflammatory bowel disease.

*Paenalcaligenes hominis*, a Gram-negative bacterium of the family *Alcaligenaceae* still largely unknown, was first described in 2010 following its isolation from the blood culture of an 85 year-old man^11^ and has been increasingly detected in the feces of elderly adults and aged mice, prompting its study in the context of neurodegenerative disorders and gut dysbiosis. As a matter of fact, *P. hominis* and *Escherichia coli*, isolated from older populations, have been implicated in colitis and cognitive decline disorders like Alzheimer’s Disease. It has been proposed that *P. hominis* extracellular vesicles exacerbate colitis-associated cognitive decline by reaching the brain through the bloodstream and vagus nerve.^11^ Supporting this, fecal transplants from aged donors (humans and mice) containing *P. hominis* induced more severe colitis and significantly greater cognitive impairment in younger recipients compared to transplants from younger donors.^12^ This connection highlights the significant impact of specific gut microbes on neurological health and disease progression through mechanisms that affect both gut and brain health.

In this context, the innate immune system comes into play distinguishing between intrusive pathogens and harmless commensals of the intestinal microbiota to prevent the presence of external agents that could cause dysbiosis of the GBA.^13–16^ Innate recognition depends on the detection of conserved microbial structures, known as “microbe-associated molecular patterns” (MAMPs), to initiate responses.^17,18^ Lipopolysaccharides (LPSs), primary components of Gram-negative bacterial outer membranes, are critical MAMPs, triggering intracellular signaling that governs infection, inflammation, and symbiosis. LPS features a tripartite architecture: the glycolipid portion, called lipid A, and a heteropolysaccharide made up of the core oligosaccharide (core OS) and the O-polysaccharide portion (or O-antigen) (Scheme S1).^19^ The structural and functional characterization of LPS from microbiota, whose role in health and disease remains debated, particularly within GBA as in the case of *P. hominis*, is a crucial area of study. Gaining deeper insights into these LPS molecules could significantly enhance our understanding of their potential involvement, not only at the molecular level but also in the broader context of bacterial contributions to the delicate balance between health and disease. This is especially relevant for aged individuals colonized by *P. hominis*, where its impact on host physiology remains unclear. We here present the comprehensive structural characterization of *P. hominis* LPS. This characterization was achieved through a combination of chemical, spectroscopic, spectrometric and computational methods. Additionally, we performed biophysical analyses, including static and dynamic light scattering (DLS) and neutron reflectometry (NR), on *P. hominis* LPS aggregates and LPS-containing biomembranes alongside investigations into its immunological properties and innate immune lectin recognition.

Given that *P. hominis* extracellular vesicles have been implicated in colitis-associated cognitive decline, we focused on elucidating the structural features of their principal components, namely, the LPS mixture, which may mediate such host–microbe interactions. Although this study does not establish causality, and acknowledging that, in the context of the gut microbiota, LPS represents a heterogeneous ensemble rather than a single molecular entity, our findings provide a molecular framework for understanding the immunological properties of *P. hominis* LPS. This work lays the foundation for future investigations into its potential role in aging-associated neuroinflammation and host immune modulation.

## Results

### LPS Isolation and Chemical Analyses

*The P. hominis* LPS mixture was isolated from dried bacterial pellet and purified with DNase, RNase, and proteases, followed by dialysis, ultracentrifugation, and gel filtration chromatography; in addition, the repurification protocol by Hirschfeld et al.^20^ was performed to remove any traces of lipoproteins possibly contaminating the isolated LPS. SDS-PAGE analysis (Figure S1A) with silver nitrate staining confirmed the smooth nature of the *P. hominis* LPS mixture, revealing discrete bands at different molecular weights in the upper and medium parts of the gel, consistent with the mass spectrometry (MS) results (see below). By contrast, no bands were visible in SDS-PAGE analysis, followed by Coomassie Brilliant Blue gel staining, indicative of the absence of any contaminating protein/lipoprotein in the isolated LPS material. Compositional analyses (Figure S1B) highlighted mainly the occurrence of l-rhamnose (L-Rha) and d-Glucosamine (d-GlcN), 3-*O*-methyl-l-rhamnose (Rha3OMe), and in minor amount d-mannose (d-Man), d-galactose (d-Gal), l-*glycero*-d-*manno*-heptose (l,d-Hep), and 3-deoxy-d-*manno*-oct-2-ulosonic acid (Kdo). Methylation analysis showed the presence of terminal, 2-substituted and 3-substituted Rha (t-Rha, 2-Rha, 3-Rha), 4-glucosamine (4-GlcN); in minor abundance 3-Hep, 3-Man, t-Gal, 3,4-GlcN, 5-Kdo and t-Kdo. As for fatty acid analysis, dodecanoic acid (12:0), tetradecanoic acid (14:0), (*R*)-3-hydroxydodecanoic [12:0 (3-OH)], (*R*)-3-hydroxytetradecanoic [14:0 (3-OH)] acid, and hexadecanoic acid (16:0) were identified.

### LPS O-Antigen Isolation and Characterization

To characterize the O-antigen, an aliquot of pure LPS underwent *O*-deacylation and was then further purified via gel permeation chromatography. The *O*-deacylated LPS (**LPS**_**OdeAc**_) was subjected to an extensive NMR investigation to assign all spin systems and to establish the saccharide sequence (Figure 1 and Table S1). The anomeric configuration of each monosaccharide was determined based on the ^3^ *J*_H1,H2_ coupling constants, whereas vicinal ^3^ *J*_H,H_ ring coupling constants led to the identification of the configuration of each sugar residue. Finally, combined information derived from *inter*-residue NOE contacts and heteronuclear long-range correlations, together with compositional analysis, allowed for the complete characterization of the *P. hominis* O-antigen.

**Figure 1:**
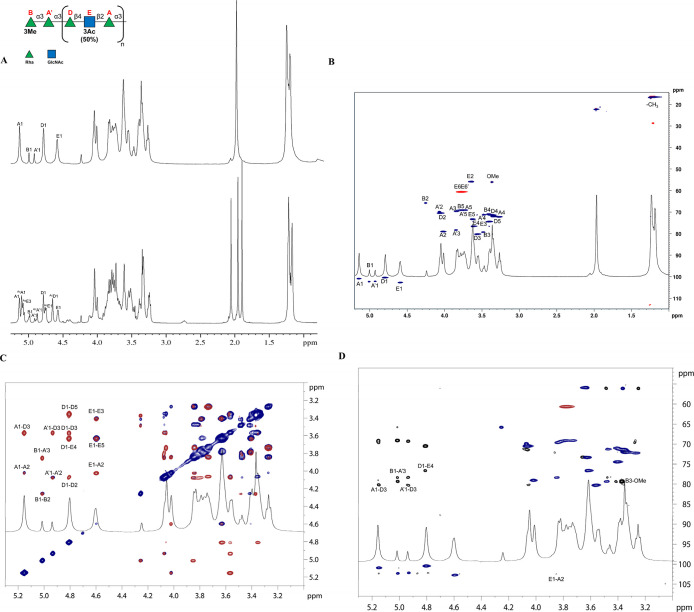
(A) ^1^H NMR spectra of O-deacylated LPS (**LPS**_**OdeAc**_, top) and of O-antigen (**OS**, bottom) form *P. hominis* LPS (schematic representation with SNFG). Anomeric signals are expanded in the inset and attributed in Table S1 (B) Zoom-in of the multiplicity edited heteronuclear single-quantum coherence (HSQC) spectrum (blue and red), highlighting the assignment of proton–carbon correlations for individual sugar residues. (C) Overlay of NOESY (red) and TOCSY (blue) *P. hominis* **LPS**_**OdeAc**_ spectra, used to identify both intraresidue proton connectivities and inter-residue NOEs crucial for establishing the monosaccharide sequence and linkage positions. Key *intra*- and *inter*-residue cross-peaks are labeled. (D) Superimposition of HSQC (blue/red) and heteronuclear multiple–bond correlation (HMBC) (black) from *P. hominis* **LPS**_**OdeAc**_ spectra, providing short- and long-range heteronuclear correlations, respectively, essential for confirming anomeric configurations and interglycosidic linkages. Sugar residue assignments correspond to letters defined in Table S1.

NMR spectra revealed the presence of 5 sugar units, all present as pyranose rings, according to both ^13^C chemical shift values and long-range correlations among positions 1 and 5 present in the HMBC spectra (Figure 1, Table S1). Spin systems **A**, **B**, **A′** and **D** were identified as Rhamnose residues, as attested by the scalar correlations of the ring protons with the methyl signal at position 6, visible in the TOCSY spectrum. The *manno* configuration was established by ^3^ *J*_H‑1,H‑2_ and ^3^ *J*_H‑2,H‑3_, both below 2 Hz and diagnostic of the H-2 equatorial orientation. The α-anomeric configuration of **A**, **B,** and **C** was assigned with the ^1^ *J*_CH_ coupling constant values (Table S1). The β-anomeric of **D** was assigned with ^1^ *J*_CH_ values and the *intra*-residual NOE contact between H-1, H-3, and H-5. Finally, spin system **E** was assigned to a β-GlcNAc; the *gluco* configuration was indicated by the ring ^3^ *J*_H,H_ coupling constants, while in the HSQC spectrum, H-2 resonance correlated to a nitrogen bearing carbon signal at 56.1 ppm. The *intra*-residual NOE contact of H-1 with H-3 and H-5 and the ^1^ *J*_CH_ values were indicative of β–anomeric configuration (see also Table S1).

The downfield displacement of the carbon resonance for **B3** was ascribable to the presence of a methoxy group, as proven by the NOE and long-range correlations of H-3 **B** with the corresponding *O*-methyl group. The glycosylated positions of the sugar units were identified with downfield-shifted carbon resonances at **A2**, **A′3**, **D3**, and **E4**; **B** was identified as a terminal sugar unit. Both *inter*-residual NOE contacts and long-range correlations in the HMBC spectrum allowed to determine the structure of *P. hominis* LPS O-antigen.

The O-antigen repeating unit was composed of residues **A**, **D**, and **E**, connected as follows [−2αRha-3βRha-4αGlcNAc−]_*n*_ (see also Figure 2). Noticeably, a terminal cap, located on the nonreducing end of the saccharide chain, was identified in the terminal αRha3OMe unit (**B** residue, Figures 1 and 2 and Table S1), located at O-3 of the α-Rha **A′**, instead of being substituted at O-2 by a GlcNAc residue (**E′**). The structural complexity of *P. hominis* LPS was further elucidated by mild acid hydrolysis, which separated lipid A from the glycan moiety, specifically cleaving the glycosidic bond between the Kdo and the lipid A. Analysis of the NMR spectra of the isolated O-antigen (Figures 1 and S2 and Supporting Information) highlighted how the O-3 position of residue **E** was in turn not stoichiometrically acetylated (50%). NMR data corroborated the previously proposed structure and the presence of a terminal cap to the repeating unit sequence, with the final structure being **B-3A′-[3D-4E-A−]**_**n**_; **E3** is acetylated at 50% (Rha3OMe-3αRha-[3βRha-4αGlcNAc3R-2αRha−]_*n*_ (R = H or Ac (50%)).

**Figure 2:**
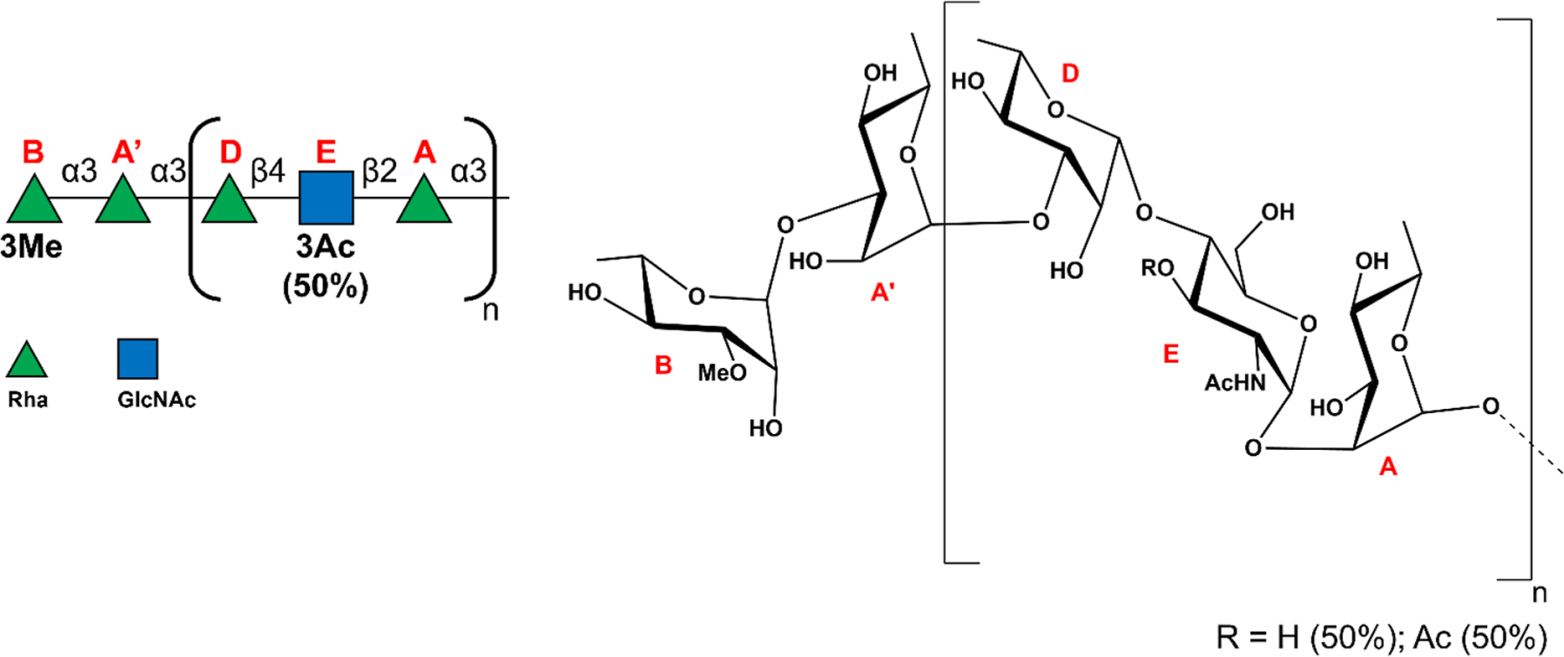
Structure of *P. hominis* LPS O-antigen.

### MS Structural Characterization of LPS and Lipid A

An in-depth negative-ion MALDI-TOF MS and MS/MS investigation was performed on isolated lipid A (Figure 3). As shown in Figure 3, the spectrum revealed the presence of several peaks in the mass range *m*/*z* 1261.5–1585.6, which were assigned to lipid A species with a different acylation and phosphorylation degree. Mass differences of 14 amu (−CH_2_– unit) or 28 amu (−(CH_2_)_2_– unit), attributable to lipid A differing by the length of their acyl chains, were also detected for some peaks (Figure 3). Briefly, and based on the compositional analysis, the main peak at *m*/*z* 1505.6 matched with a *mono*-phosphorylated penta-acylated lipid A carrying four primary 14:0 (3-OH) and one secondary 12:0. Tetra-acylated lipid A species devoid of a 14:0 (3-OH) or 12:0 ratio were also detected at *m*/*z* 1279.5 and *m*/*z* 1323.5, respectively. Likewise, related *bis*-phosphorylated lipid A forms were attributed to peaks at *m*/*z* 1585.6, *m*/*z* 1403.5, and *m*/*z* 1359.5 (Figure 3). Negative-ion MS/MS investigation conducted on several ion peaks allowed for the location of both the secondary acyl substitution and the phosphate in *mono*-phosphorylated lipid A species. In Figure S3 is reported, as an example, the MS/MS spectrum of precursor ion at *m*/*z* 1279.5 attributed to a *mono*-phosphorylated lipid A species carrying three 14:0 (3-OH) and one 12:0. The concomitant presence of specific ion peaks, such as those at *m*/*z* 994.4 (^0,3^A_2_), *m*/*z* 934.4 (^0,4^A_2_), and *m*/*z* 812.4 (^0,2^A_2_-12:0),^21^ originating from the cross-ring fragmentations occurring on the reducing glucosamine, enabled to locate both the phosphate and the secondary 12:0 on the nonreducing glucosamine. In addition, the absence of an ion derived by the loss of a whole unit built of one 14:0 (3-OH) and 12:0 guided the positioning of the latter on the primary amide-bound fatty acid.

**Figure 3:**
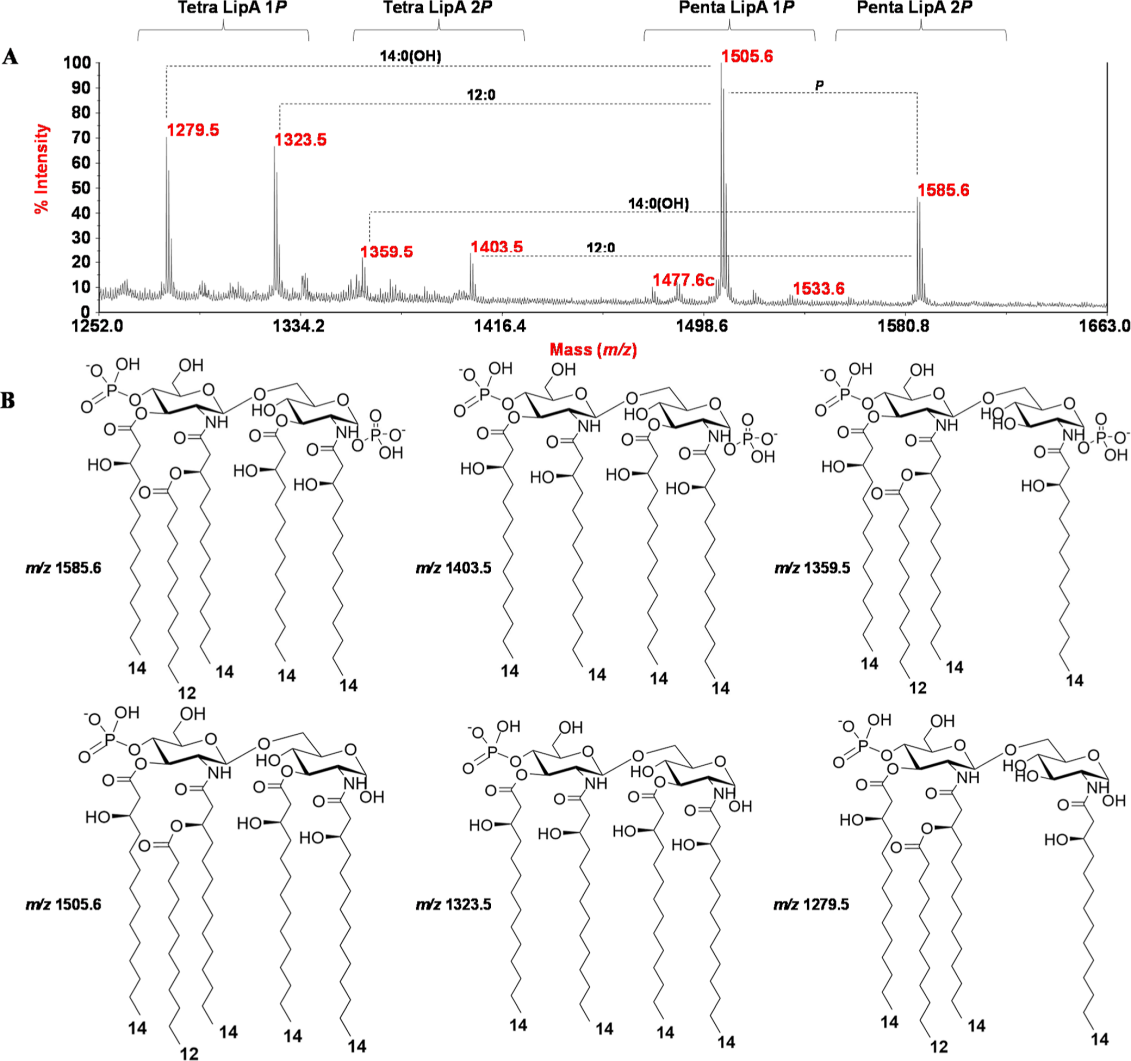
(A) Negative-ion MALDI-TOF MS spectrum, recorded in reflectron mode, of lipid A fraction from *P. hominis* LPS. (B) Proposed structures of main *mono*- and *bis*-phosphorylated lipid A species.

In parallel, an aliquot of LPS underwent hydrolysis with diluted ammonium hydroxide to achieve selective *O*-deacylation, followed by negative-ion ESI-MS analysis. The ESI-MS spectrum is shown in Figure 4A, with the main observed ions listed along with the proposed structures in Table S2. Direct infusion of *O*-deacylated LPS (LPS_deOAc_) produced many ions attributed to intact LPS_deOAc_, as well as Y-type (related to lipid A) and B-type (related to the carbohydrate portion) fragments thereof. The major deprotonated molecular ions related to intact LPS_deOAc_ were detected in the quadruply (M–4H)^4–^ or quintuple (M–5H)^5–^ charged states, while ions corresponding to lipid A and the carbohydrate portion were mainly detected as singly or triply charged species. Strikingly, this approach provided insights into the core oligosaccharide (OS) structure and highlighted the structural heterogeneity of *P. hominis* LPS, as proven by SDS-PAGE analysis (Figure S1A). Minimum core OS, detected at *m*/*z* 1230.42, was assigned to a heptasaccharide consisting of one anhydrous Kdo, one heptose, one *N*-acetyl-hexosamine (HexNAc), two deoxyhexoses (dHex), and two hexoses (Hex). The core OS structure was elucidated through negative-ion MS/MS (Figure 4B). The resulting data offered multiple pieces of evidence, illustrated in the inset of Figure 4B, which guided the establishment of the primary structure. A related ion at *m*/*z* 1272.43 was assigned to the same core OS carrying an additional acetyl group on the HexNAc residue (Figure 4A). In the higher *m*/*z* range, intense peaks ascribable to (M - 5H)^5–^ ions corresponding to LPS_deOAc_ were detected, with the most intense one at *m*/*z* 1622.88 matching with the *mono*-phosphorylated lipid A diglucosamine backbone carrying two 14:0 (3:OH) acyl chains and one 12:0, along with a core OS made up of two Kdo, two Hex, two dHex, and one HexNAc, 10 repeating units (OAg and OAg_Ac_) of the O-antigen (half of which carries an additional acetyl group) and a capping trisaccharide consisting of two dHex and one *O*-methylated-dHex, in agreement with NMR analysis. Likewise, the peak at *m*/*z* 1745.18 matched with a (M–4H)^4–^ ion corresponding to LPS_deOAc_ made up of the *bis*-phosphorylated lipid A diglucosamine backbone carrying two 14:0 (3-OH), the core OS described above (*m*/*z* 1230.42), eight repeating units of the O-antigen (half of which carries an additional acetyl group), and the capping trisaccharide.

**Figure 4:**
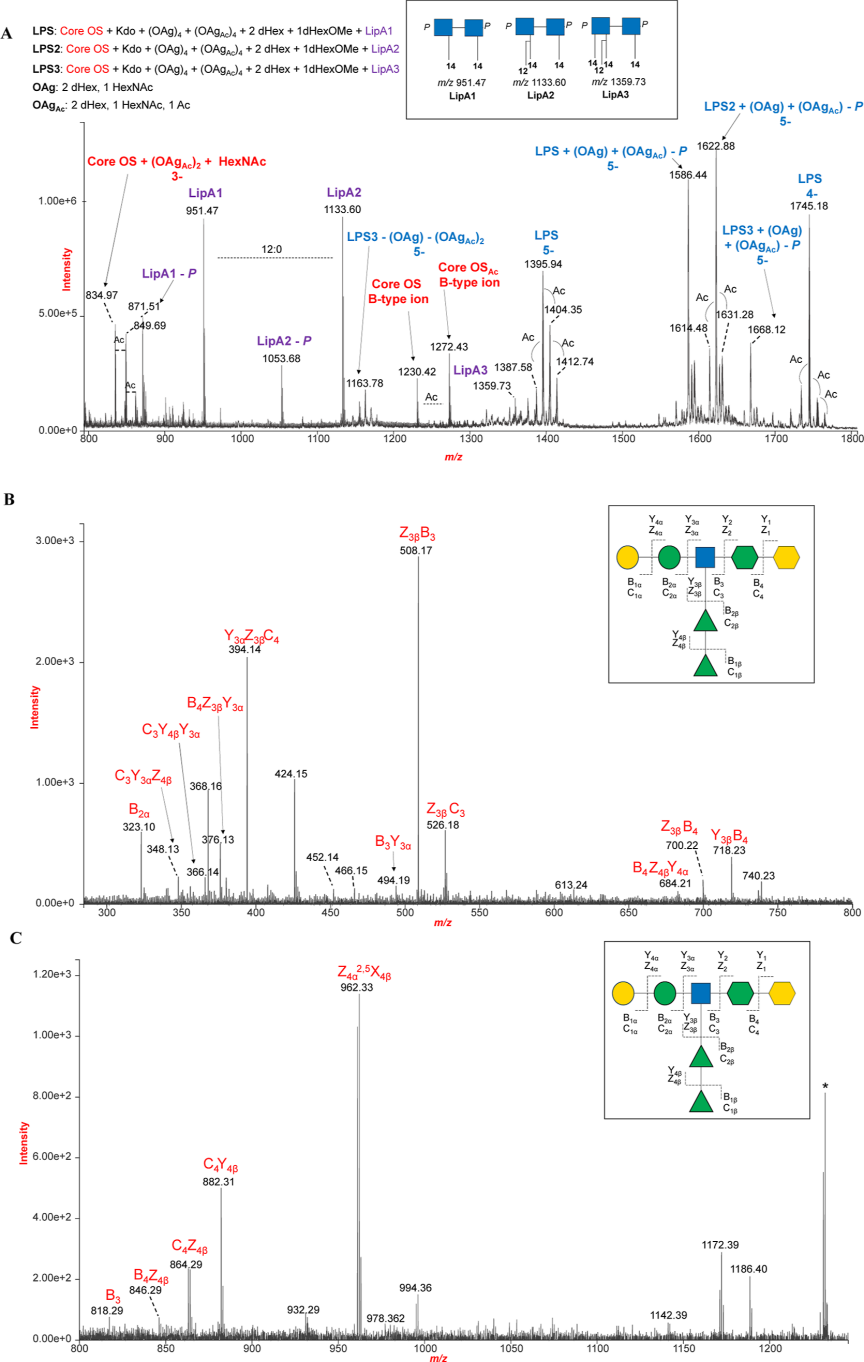
(A) Negative-ion ESI mass spectrum of the *P. hominis* *O*-deacylated LPS (**LPS**_**deOAc**_). LPS preparation yielded quadruply and quintuple charged deprotonated molecular ions for the major LPS, while mainly singly and triply charged species were detected for lipid A and core OS species. Key ions and their proposed structures are listed in Table S2. OAg_Ac_ and OAg stand for a repeating unit of the O-Antigen bearing or not an additional acetyl group, respectively. LipA stands for lipid A comprising the *bis*-phosphorylated glucosamine disaccharide backbone carrying two 14:0 (3-OH), whereas LipA2 and LipA3 bear an additional 12:0 and 12:0 and 14:0 (3-OH), respectively. A cartoon describing the *O*-deacylated positions of lipid A resulting in LipA1–3 is reported in the inset. (B,C) Enlargements of the negative-ion ESI-MS/MS spectrum of the core OS species detected at *m*/*z* 1230.42. Main singly charged ions involving cleavage of the glycoside linkage (i.e., Y, B, Z, and C type ions) were indicated in the spectrum. Unassigned peaks are related to cross-ring fragmentations in combination or not with linkage cleavage. Schematic structures of the core OS identified using MS-based characterization strategy are reported in the insets. Symbols legend: green triangle: rhamnose; green circle: mannose; yellow circle: galactose; blue square: *N*-acetyl-glucosamine; green hexagon: heptose; yellow hexagon: 3-deoxy-d-*manno*-octulosonic acid, Kdo. The structure is tentatively given according to compositional analyses.

Finally, full LPS underwent negative-ion MALDI-TOF MS analysis (Figure S4). The reflectron MALDI-TOF MS spectrum of *P. hominis* LPS showed at higher molecular masses (between *m*/*z* 5817 and 7720) a series of [M – H]^−^ ions related to the native LPS mixture differing in mass by 42 mass units, consistent with a variable content of endogenous O-acetylation of the O-antigen repeating unit, in accordance to ESI-MS analysis of the LPS_deOAc_. Other LPS species were found to differ from the main one detected at *m*/*z* 7183.9 by 537 mass units (*m*/*z* 7720.9), which was consistent with the presence of an additional repeating unit (OAg_Ac_) made up of two dHex, one HexNAc and one acetyl group, and thus in accordance with NMR and ESI MS analysis. LPS species with shorter O-antigen, i.e., displaying one or two repeating units less, were also identified (Figure S3B). Interestingly, ion fragments derived by the cleavage of the glycosidic bond between Kdo and the lipid A portion were also identified at lower mass ranges (*m*/*z* 1500–2510). This LPS fragmentation (β-elimination) yielding both lipid A and oligosaccharide (core OS) ions provided important insights into the structure of the *P. hominis* LPS. A further lipid A species, carrying three phosphates, was also identified at *m*/*z* 1666.9, not detected in MS analysis of both lipid A and O-deacylated products. In addition, the intense peak at *m*/*z* 2507.7 was attributed to the core OS, and this was supported by the observation of the twin ion peak at *m*/*z* 2463.7 (−44 amu), derived from the neutral loss of a CO2 molecule from the reducing Kdo. By merging data from ESI-MS and MALDI-TOF MS, it was proposed that the core OS at *m*/*z* 2507.7 shares the same composition as the species detected at *m*/*z* 834.96 in the ESI-MS analysis (Figure 4A), with the addition of two OAg_Ac_ repeating units and a terminal HexNAc.

Overall, the integrated MS and MS/MS analyses revealed that *P. hominis* LPS is composed of a structurally heterogeneous lipid A domain, encompassing *mono*-, *bis*-, and even *tris*-phosphorylated tetra- and penta-acylated species with varying acyl chain lengths. The core OS was defined as a heptasaccharide featuring Kdo, Hex, dHex, and HexNAc residues, occasionally carrying additional acetyl modifications. The O-antigen displayed variable chain lengths and endogenous *O*-acetylation, contributing to a range of intact LPS species differing by 42 mass units. The detection of high-mass [M–H]^−^ ions alongside diagnostic fragment ions provided strong evidence for a complex and microheterogeneous LPS architecture, consistent with NMR data and potentially indicative of a finely tuned immunomodulatory potential.

### Computational Studies

The three-dimensional architecture of *P. hominis* O-antigen was described by computational approaches.^22^ In detail, the disaccharide units contained in the O-antigen were constructed and the energetically accessible conformational regions were investigated by MM calculations. The corresponding adiabatic energy maps for the glycosidic torsions Φ (H1–C1–O–CX′) and Ψ (C1–O–CX′–HX′, Figure S5) were used to build the O-antigen and run molecular dynamics (MD) simulation in explicit solvent with AMBER. To investigate the effects of nonstoichiometric acetylation of **E** residue, computational studies were conducted with glycans in both *O*-deacetylated and fully acetylated states. MD simulations were performed on an octasaccharide, comprising a terminal tetrasaccharide (**B-A′-D-E**) and a repeating trisaccharide (**A-D-E**), followed by cluster analysis to determine its overall structural features and conformational preferences. The most representative conformers (Figure 5) shared comparable shapes and side. The glycosidic linkages adopted Φ values in accordance with the *exosyn* anomeric conformation (Figures 5 and S5).^23^ Representative conformers showed how the O-antigen possessed a slight flexibility around ψ torsion angles (oscillating form −20° to 20°) and a loose coiled shape (Figure S6). Moreover, the presence of acetyl groups had no significant impact on the structure, with the superposition of both ligands demonstrating consistent trends over the 200 ns MD simulation period.

**Figure 5:**
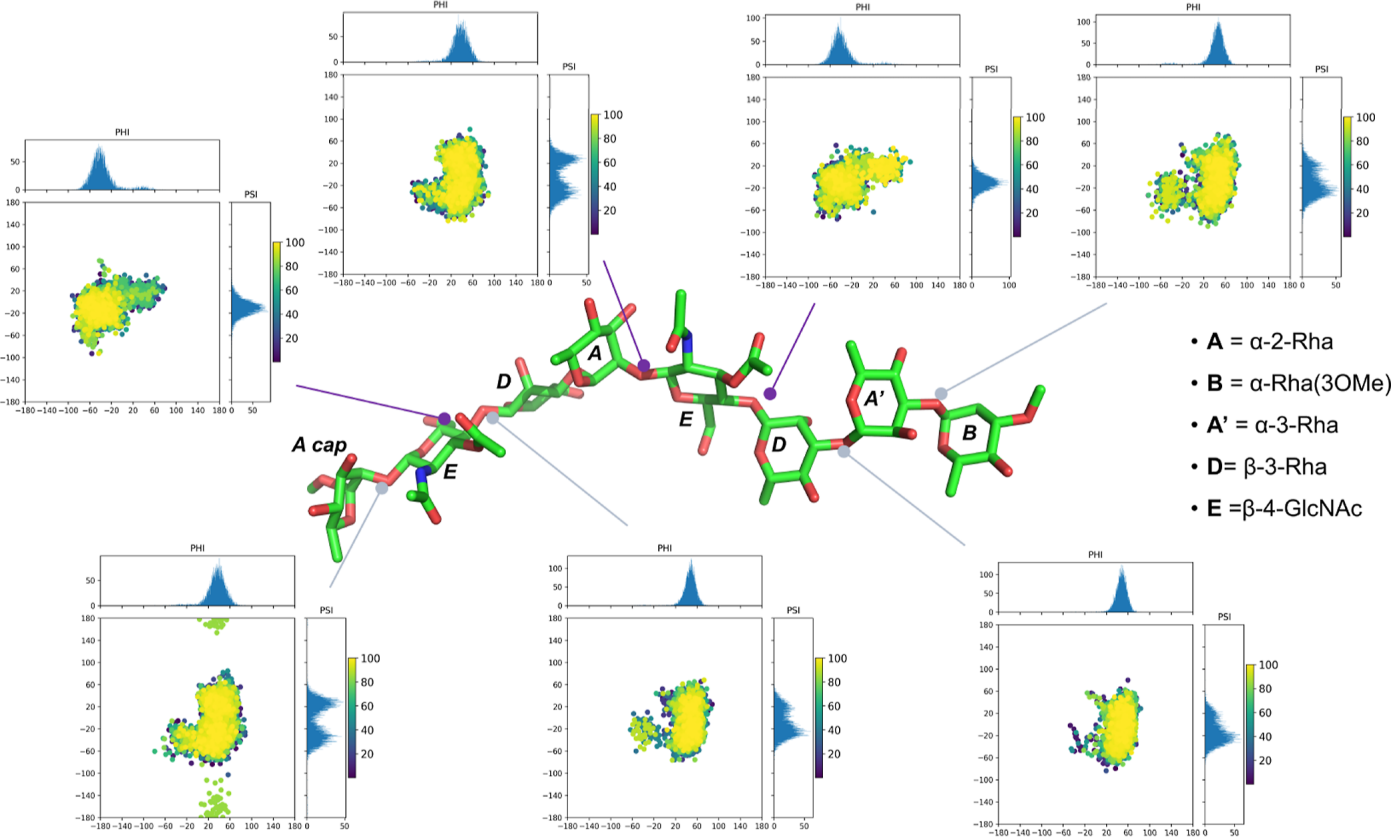
Torsion angle distributions around the glycosidic linkages throughout the MD simulation of *P. hominis* **LPS**_**OdeAc**_ octasaccharide. The torsion angles (Φ and Ψ) were monitored for each glycosidic bond along the entire MD simulation. The two-dimensional plots display the distribution of torsion angles for each linkage, with the color gradient representing the frequency of each angle occurrence. The **LPS**_**OdeAc**_ octasaccharide is shown in its most representative conformation, with each sugar residue labeled according to Table S1.

For a more comprehensive understanding of the three-dimensional behavior of the antigen phenotype as a function of length, computational studies were carried out using longer glycans, specifically containing the terminal tetrasaccharide (**B-A′-D-E**) and three repeating trisaccharide units (**A-D-E**)_3_ and modeled in both acetylated and nonacetylated forms; consistent with previous findings, they exhibited similar behavior, indicating that acetylation of **E** did not significantly alter the 3D properties, of the O-antigen 3D properties, with the only slight difference being that the nonacetylated compound was more compact (Figures 6 and S7).

**Figure 6:**
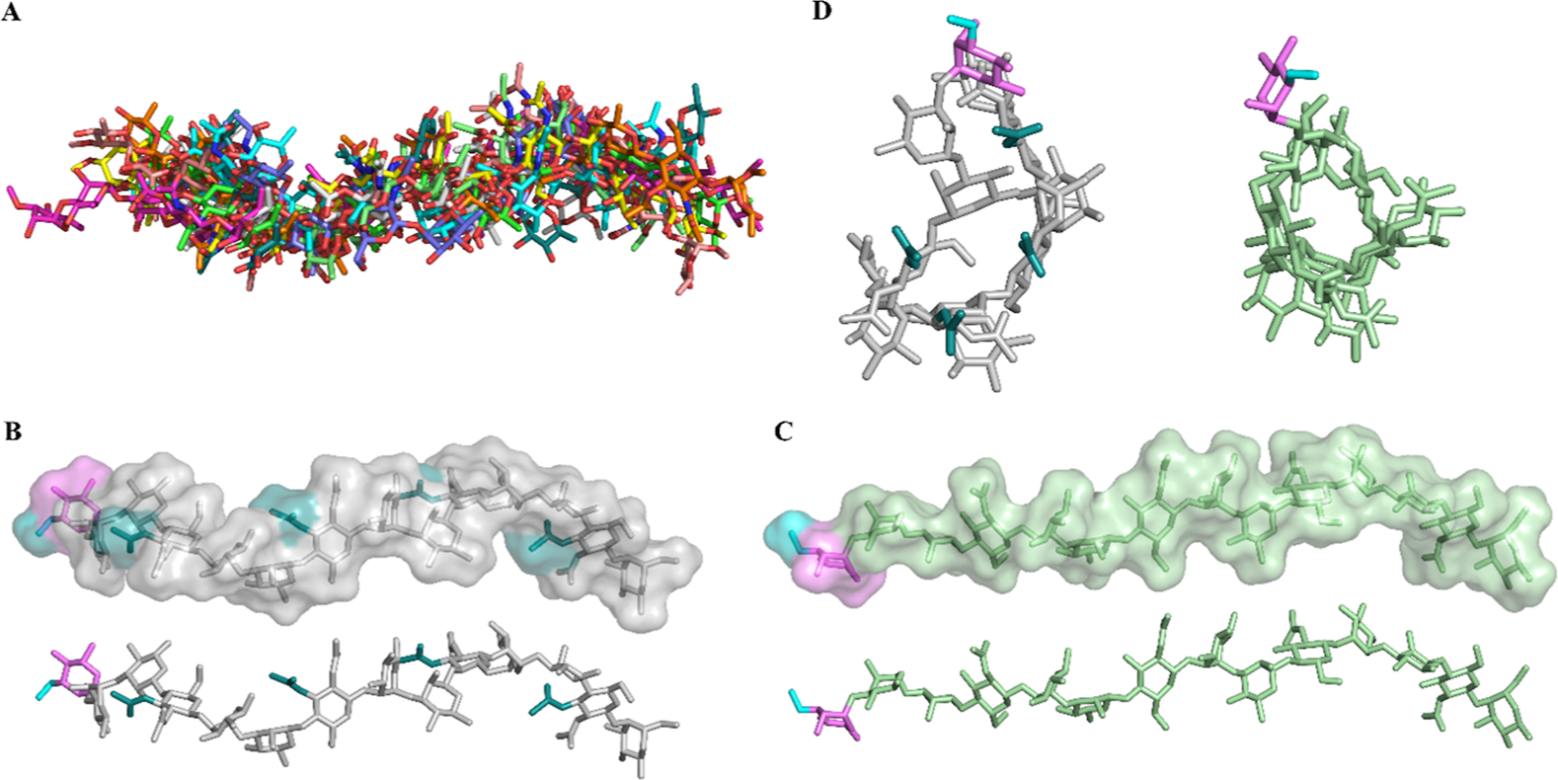
Computational study of the MD of *P. hominis* O-antigen and **LPS**_**OdeAc**_ tetradecasaccharides. (A) Superposition of all poses obtained during the MD simulation of the **O-antigen** tetradecasaccharide. (B) Most representative pose of the O-antigen tetradecasaccharide (white), highlighting acetyl groups in teal. The terminal sugar (**B** residue) is highlighted in pink, with its terminal OMe group shown in cyan, displayed both with and without the molecular surface representation. (C) Most representative pose of **LPS**_**OdeAc**_ tetradecasaccharide (green), also highlighting the terminal sugar (pink) and its OMe group (cyan), shown with and without surface. (D) Front view of O-antigen (white) and **LPS**_**OdeAc**_ (green) tetradecasaccharides, emphasizing their similar helical conformations.

### Immunological Assays

We then conducted an immunological evaluation of *P. hominis* LPS. Stimulation of HEK-Blue cells transfected with LPS-recognizing human molecular complex TLR4, MD-2, and CD14 genes with different concentrations of *P. hominis* LPS (1, 10, and 100 ng/mL) was performed. *E. coli* serotype OIII:B4 LPS is well-known to act as a potent agonist on the TLR4-MD2 receptor, so it was used as the positive control at the same concentrations. Activation of NF-kB was the readout of this experiment (Figure 7A). The results showed that *P. hominis* LPS was a weaker activator of the TLR4 signaling compared to *E. coli* LPS at all concentrations tested (*P. hominis* vs *E. coli* LPS, *p*-value <0.001 at 1 ng/mL and 10 ng/mL, and *p*-value <0.01 at 100 ng/mL, Figure 7A). In parallel, *P. hominis* LPS was also used to stimulate HEK-Blue cells transfected with TLR2 and no activation was observed (Figure 7B), thus indicating that *P. hominis* LPS is unable to activate TLR2 signaling. Furthermore, the ability of *P. hominis* LPS to stimulate the production of inflammatory cytokines by peripheral blood mononuclear cell lines (PBMCs) was also evaluated. The simultaneous analysis of different secreted inflammatory cytokines performed on blood from six healthy donors (three male and three female) showed that *P. hominis* LPS was able to cause an increase in the concentration of TNF-α and IL-10 with the concomitant significant reduction of the concentration of IL12p70 and IL-17A, compared to unstimulated cells (Figure S8). Collectively, these preliminary results indicate that the *P. hominis* LPS mixture elicits a weak TLR4-mediated response, does not activate TLR2, and induces only a modest and cytokine release profile in PBMCs, supporting its characterization as a low-inflammatory, potentially immunomodulatory LPS pool.

**Figure 7:**
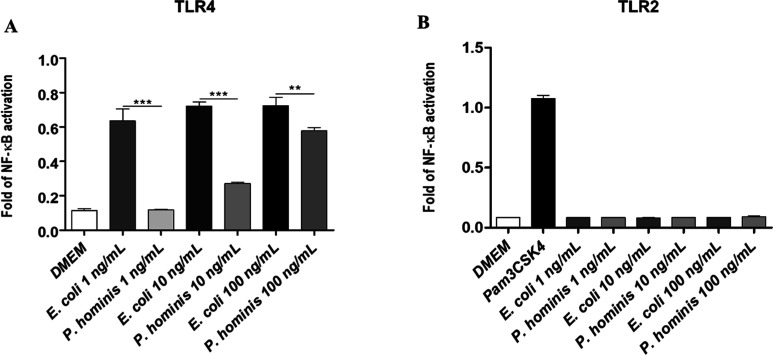
TLR activation by *P. hominis* LPS. (A) Stimulation of HEK Blue hTLR4 and (B) HEK Blue hTLR2 cells. Secreted embryonic alkaline phosphatase (SEAP) levels (OD) upon stimulation with LPS from *E. coli* and *P. hominis* at the indicated concentrations (1, 10, 100 ng/mL). Ultrapure LPS from *E. coli* 0111:B4, a potent TLR4 agonist, was used as a positive control in (A), whereas in (B), it was used as a negative control. The positive control in (B) was Pam3CSK4 (500 ng/mL), a synthetic triacylated lipopeptide agonist for TLR2. Significant difference between *P. hominis* LPS values and the corresponding *E. coli* LPS (*P. hominis* vs *E. coli*, *** *p* < 0.001, ** *p* < 0.01) by the Student *t*-test are indicated. Data are expressed as mean ± SD of three independent experiments.

### Microarray Studies

A microarray analysis was conducted using two distinct preparations of the *P. hominis* LPS: Mix1, a mixture containing both long and short O-antigen forms, and Mix2, enriched in the shorter O-antigen form and exhibiting a significant abundance of the core oligosaccharide region. The aim was to evaluate the binding affinity of various lectins and immune-related proteins, including galectins and ficolins, among others. Surprisingly, meaningful binding signals were detected for galectin-4, despite the absence of a galactose residue in the *P. hominis* structure, with an apparent preference for the fraction enriched in the shorter O-antigen form. Recognition of the galactose residue present in the core OS could account for this behavior. In contrast, ficolin-3, as well as intelectin-1 and mindin, showed a preference for Mix1 over Mix2 (Figure 8), while Ficolin-2 exhibited a similar binding intensity to both preparations. These results suggest differential recognition patterns between the LPS forms, which require further investigation.

**Figure 8:**
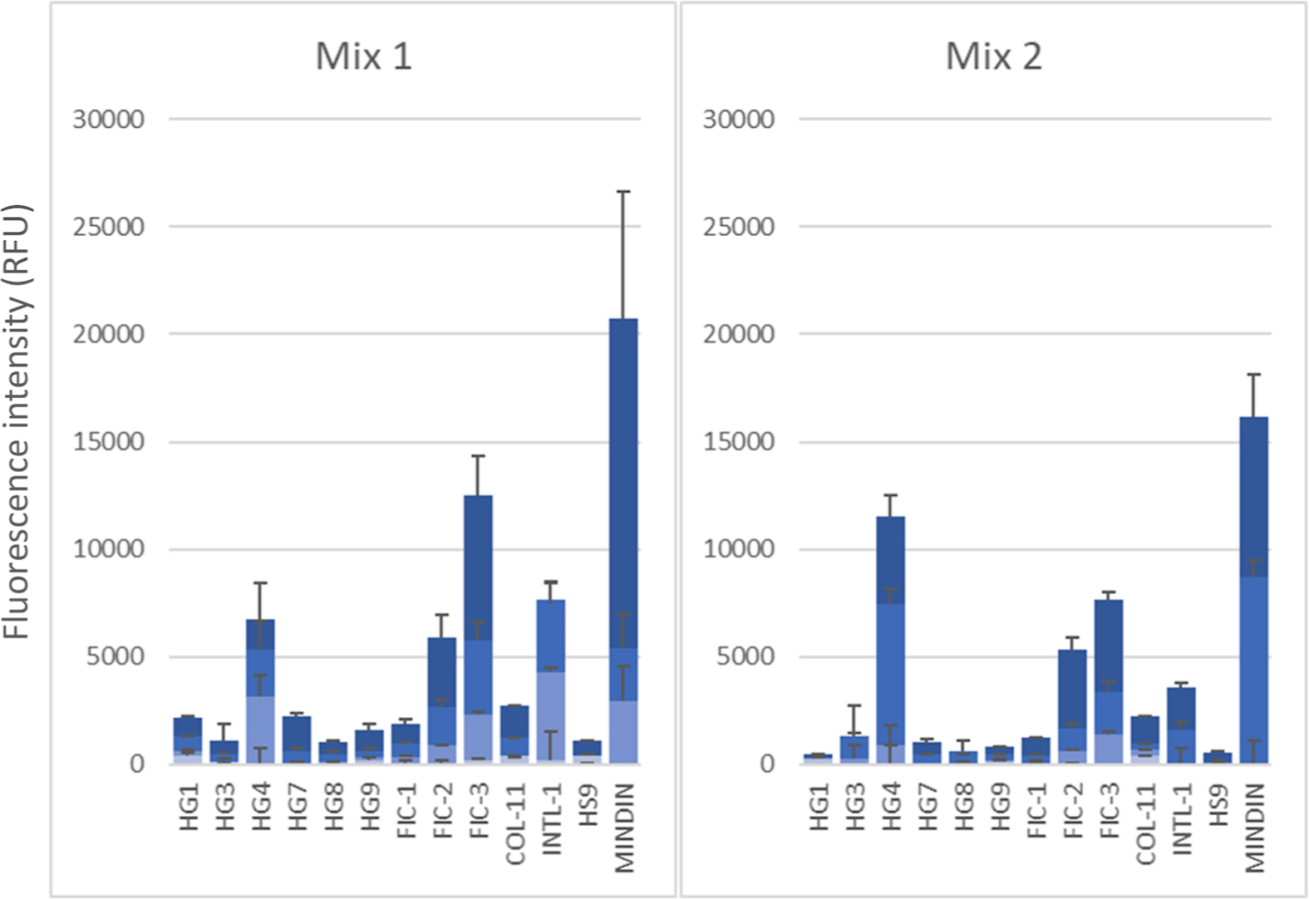
Microarray analysis of *P. hominis* LPS recognition by various lectins and immune-related proteins. Two different LPS preparations, Mix1 (containing both long and short O-antigen forms) and Mix2 (enriched in the shorter O-antigen form), were printed as duplicates at different concentrations and the binding of the different proteins was assayed using AF647-streptavidin for final detection. Data shown correspond to the mean of the fluorescence signals obtained for samples printed at 1, 0.3, 0.1, and 0.03 mg/mL (depicted in dark to light blue color scale), and error bars indicate the standard deviation of the mean. HG, human galectin; FIC, ficolin; COL, collectin; INTL, intelectin; HS, human Siglec.

### Characterization of LPS Aggregation and of Asymmetric LPS-Containing Bilayers Using DLS, SLS, and Neutron Reflectometry

DLS and SLS experiments allowed us to determine the hydrodynamic radius, molecular mass, and number of LPS involved in the aggregation process.^24,25^ DLS analysis revealed the formation of a single LPS aggregate characterized by an *R*_h_ = 30 ± 2 nm (Figure 9A). The observed monomodal hydrodynamic radius distribution enabled us to execute SLS experiments. As depicted in Figure 9B, these experiments yielded a molecular weight of 430 ± 10 kDa for the LPS aggregate. Considering the *P. hominis* LPS estimated average molecular weight of around 7 kDa (Figure S4), the aggregate comprises approximately 61 units.

**Figure 9:**
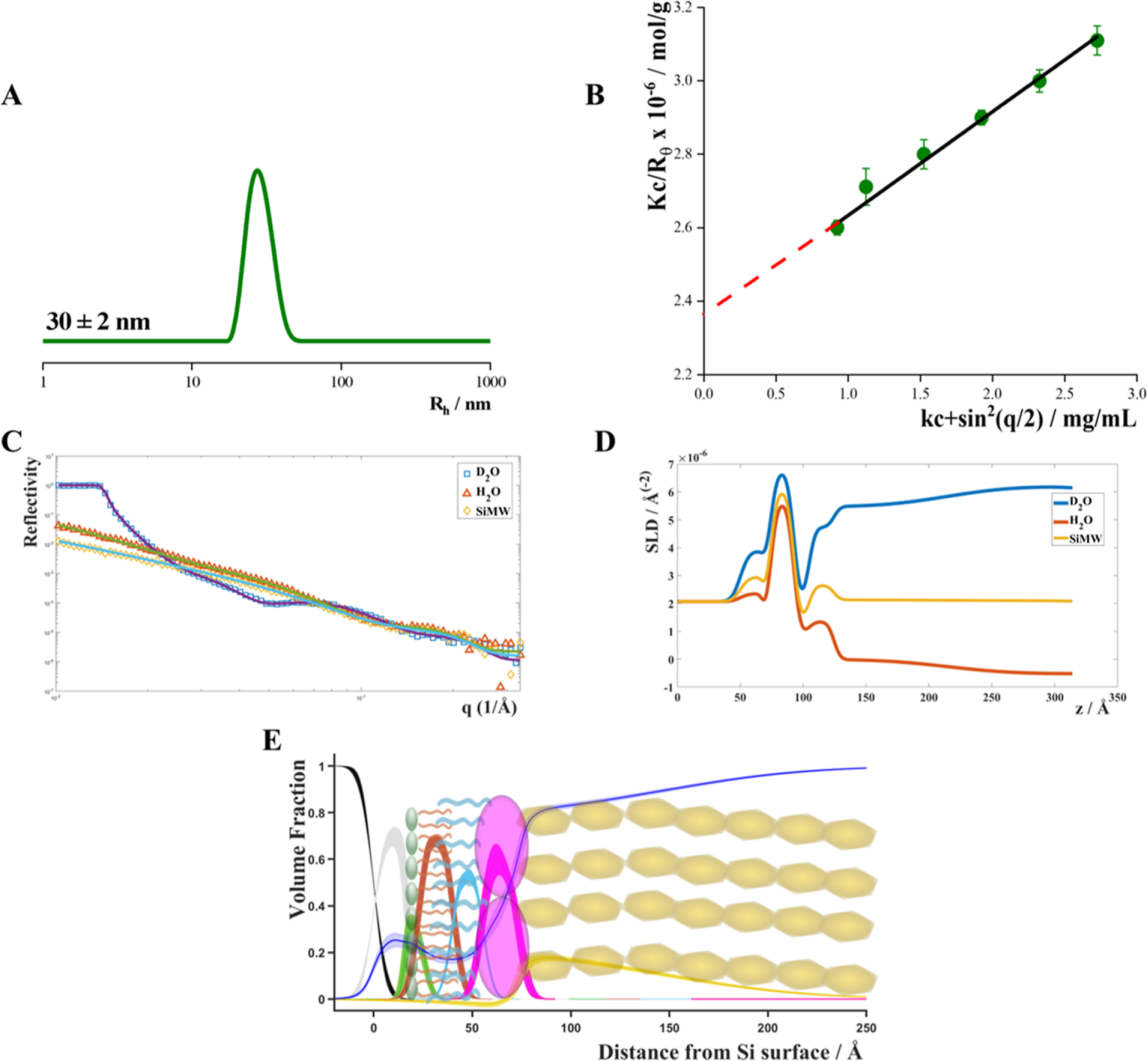
(A) Hydrodynamic radius distribution of *P. hominis* LPS, (B) Zimm plot of *P. hominis* LPS, (C) NR data with the corresponding fits, (D) scattering length density (SLD) profiles, and (E) volume fraction occupancy of *P. hominis* LPS moieties: silicon (black), silicon dioxide (gray), inner head groups (light green), PC tails (orange), LPS tails (purple), core region (midgreen), O-Antigen region (dark green).

The NR study of the asymmetric bilayer composed of d-DPPC (inner leaflet) and *P. hominis* LPS (outer leaflet) deposited onto silicon substrates^26^ was crucial for obtaining nanoscale microstructural information on LPS-containing biomembranes.

We used three contrasts to obtain different reflectivity profiles and constrained them to fit a single profile for the layer thickness and roughness. The combined analysis of the NR results, shown in Figure 9 and detailed in Table S3, demonstrated the effectiveness of our approach in achieving a highly asymmetric bilayer. As expected, the inner leaflet was primarily composed of d-DPPC, while the outer leaflet was mainly composed of *P. hominis* LPS. This assessment of sample quality was crucial to validating the fit parameters obtained.

Specifically, the inner headgroup was 6 ± 1 Å thick with 30% hydration. The lipid bilayer, consisting of the d-DPPC carbon chain and the lipid A carbon chain, was approximately 20.3 Å thick, with 16% hydration. This region of the lipid bilayer exhibited a roughness of 4 Å, indicating a high level of packing among the d-DPPC carbon chains and the *P. hominis* LPS lipid A carbon chains. Additionally, the core region was found to be approximately 20 Å thick. The core region was characterized by a thickness of approximately 20 Å and a hydration level of about 26%.

The O-Antigen region was characterized by a thickness of 90 Å and an 81% hydration level, indicating significant water penetration within this layer. The roughness of this layer was approximately 42 Å, suggesting a relatively loose packing of the O-Antigen moiety.

## Discussion

The presence of *P. hominis* in the gut microbiota of elderly individuals, along with its association with cognitive decline and colitis, suggests a potential pathogenic role for this bacterium. Therefore, identifying the structural components responsible for its (putative) virulence is essential. In this perspective, the characterization of *P. hominis* LPS adds a significant piece to the puzzle of host–microbe interactions in the gut. This study defines the distinctive structural features of *P. hominis* LPS that may contribute to its immunological properties. As a matter of fact, a deeper understanding of the LPS O-antigen structure and dynamics provides insights into its biological functions and paves the way for therapeutic strategies against its pathogenic effects in neurodegenerative and gastrointestinal disorders. We found that the O-antigen is composed of repeating units of rhamnose and glucosamine, with a terminal 3OMe-αRha (**B**, Figure 2) cap, and that nonstoichiometric acetylation of GlcNAc **E** at O-3 adds structural complexity. In-depth MS analysis of the intact and *O*-deacylated LPS mixture confirmed this structural characterization and offered new insights into the architecture of core OS and lipid A (Figures 3 and S4). Computational studies elucidated the O-antigen conformational behavior. MD simulations, using both *O*-deacetylated and fully acetylated glycans, revealed that the O-antigen structural integrity and flexibility are unaffected by acetylation. Notably, the adopted loose coiled conformation aligns with the structural and conformational features of many bacterial glycans, a shape known to promote interactions with host immune receptors and tissues.

Light scattering analyses elucidated the aggregation behavior of the *P. hominis* LPS mixture, revealing the formation of stable aggregates of approximately 61 LPS units, which may play a role in its interaction with host cell membranes and immune receptors, potentially modulating immune responses and amplifying pathogenic processes. Neutron reflectivity studies further provided nanoscale insights into the structural organization of asymmetric LPS-containing bilayers. Our results highlighted remarkable water penetration within the LPS layer and a relatively loose packing of the O-antigen portion. The observed asymmetry, with a well-defined inner and outer leaflet composition, closely mimics the natural biological membranes where LPSs reside. The detailed characterization of layer thickness, roughness, and hydration levels in the bilayer offers crucial information on how these structures may behave in vivo, potentially influencing interactions with host cells or contributing to pathogenic mechanisms.

We also investigated the impact of purified LPS species on the human innate immune system by using HEK cells transfected with TLR4 or TLR2. We showed that *P. hominis* LPS mixture activates TLR4 signaling, although to a lesser extent compared to *E. coli* LPS, a well-known potent TLR4 agonist. The *P. hominis* lipid A structure, specifically the portion recognized by the TLR4/MD-2 complex, explains these results.^27^ Indeed, hypo-acylated lipid A molecules (lipid A molecules with fewer than six acyl chains) generally activate the TLR4-mediated immune response only weakly.^28^ The penta-acylated nature of *P. hominis* LPS lipid A is the likely reason for its weaker TLR4 agonistic activity compared to the hexa-acylated *E. coli* LPS. This difference in activity occurs despite *P. hominis* LPS having acyl chains of the same length as those of *E. coli* LPS (i.e., 14 and 12 carbon atoms). Moreover, *P. hominis* LPS induces a moderate immune response also in PBMCs, characterized by a slight increase in TNF-α and IL-10, while downregulating IL-12p70 and IL-17A compared to the negative control. This suggested that it does not strongly promote Th1 or Th17 polarization and may have an immunomodulatory role rather than a highly pro-inflammatory effect (Figure S8). These results, although only preliminary, further support our hypothesis that a crucial role might be played by the carbohydrate portion of this heterogeneous LPS mixture in the pathogenicity of *P. hominis*.

Indeed, glycan-lectin microarray studies unveiled selective interactions between *P. hominis* LPS and innate immune lectins, including ficolins 2 and 3, galectin-4, and intelectin-1, highlighting the complexity of host–pathogen interactions mediated by immune system-LPS crosstalk. Ficolins, known to bind *N*-acetylglucosamine (GlcNAc) and various acetylated structures, play a pivotal role in activating the lectin pathway of the complement system, contributing to the host’s first line of defense against bacterial pathogens.^29^ The fact that ficolin-3 exhibits a preference for the mixed LPS form (Mix1, Figure 8) suggests that a longer O-antigen chain or potential structural differences enhance the interaction. The same behavior is observed for intelectin-1, which is known to interact with multiple microbial glycan epitopes and recognizes a broad range of bacterial species.^30,31^ In contrast, galectin-4, typically associated with the recognition of galactose-containing glycans,^32^ exhibited preference for the shorter LPS form (Mix2, Figure 8). This result is in line with the presence of Galactose in the core region. Indeed, the binding signals observed for the other galectins tested were small or even negligible. Galectin-4, which is highly expressed in the intestinal tract, contains two carbohydrate-recognition domains (CRDs) that bind diverse nonsulfated and sulfated glycans. Of note, we found that the binding of galectin-4 to *P. hominis* LPS was inhibited in the presence of galactose-3-sulfate (not shown). In addition, the N-terminal CRD can recognize a noncarbohydrate compound as cholesterol-3-sulfate, a property that is unique within the galectin family.^33^ Similarly, galectin-4 could bind to a specific epitope in *P. hominis* LPS that other tested galectins do not recognize. Greater exposure of this epitope in the shorter O-antigen form enriched in Mix2 would favor recognition. The interaction between galectin-4 and *P. hominis* LPS offers an interesting opportunity to explore new aspects of bacterial immune evasion and host–pathogen interactions, particularly in the context of gastrointestinal health and immune modulation. Finally, the repertoire of interactions between *P. hominis* LPS and innate immune lectins is further broadened by the intense binding signals observed for mindin. This is a highly conserved extracellular matrix protein that is expressed in several tissues and has multiple functions, including acting as a pattern-recognition molecule for microbial pathogens.^34,35^ Mindin was reported to interact with lipoteichoic acids from Gram-positive bacteria and with the LPS from *Salmonella typhosa* and *E. coli* through carbohydrate recognition.^34^ However, the binding of mindin to the LPS of other bacteria has not been described so far. Further investigation of the specific epitopes recognized by this lectin will help to understand its role in the innate immune response.

Our findings reveal that *P. hominis* LPS possesses unique structural and immunological properties that may shape host–microbe interactions within the gut, particularly in elderly individuals. The distinct composition of its O-antigen, along with its lipid A and core OS structure, suggests a finely tuned immune recognition mechanism that deviates from the classical pro-inflammatory response typically associated with Gram-negative bacteria. The weak activation of TLR4, coupled with selective recognition by immune lectins such as Ficolin-3 and Galectin-4, may suggest the involvement of noncanonical immune recognition routes. However, the contribution of these interactions to microbiota-driven immune modulation remains to be elucidated. While we consistently refer to the *P. hominis* LPS as a heterogeneous mixture, it is important to stress that the observed immunological properties likely arise from the combined effects of multiple coexisting LPS species. This structural diversity, typical of microbiota-derived LPS,^36–38^ may represent a key determinant of their immunomodulatory behavior. Moreover, it cannot be excluded that the lipid A and glycan composition of outer membrane vesicle-associated LPS, which seem to be connected to gut–brain axis dysfunction, differ from those of the bulk LPS pool. A particularly relevant future direction will be the isolation and characterization of LPS directly from *P. hominis* extracellular vesicles, to determine whether they differ structurally from the total LPS mixture and contribute uniquely to immune modulation.

Overall, this study offers a compelling example of how subtle structural features of the LPS can reshape host responses. As the gut–brain axis emerges as a key player in aging and neurodegeneration, deciphering such molecular dialogue will be critical. The structural features identified in *P. hominis* LPS provide a basis for exploring how microbiota-derived molecules may influence host immunity. These findings could support future efforts to design targeted interventions, although further studies are required to establish causal links. In this context, the generation and immunological testing of synthetic or semisynthetic LPS derivatives will be instrumental in dissecting structure–function relationships and identifying the specific molecular motifs responsible for immune modulation. The unique immunological fingerprint of *P. hominis* LPS opens new avenues for understanding microbiota-driven modulation of immunity and brain health, and potentially, for designing therapeutic interventions targeting microbial components in age-associated diseases.

## Methods

### Healthy Subjects and Cytokines Determination

Healthy subjects enrolled in this study were enrolled according to the protocol approved by IRCCS Pascale, Institutional Ethical Review Board (CE: Protocollo no. 4/21, 2021). For the cytokines expression evaluation, 200 ng/mL of the O-antigen of *P. hominis* was added to 5 × 10^5^ PBMC from 6 healthy donors (3 male and 3 female median age 32 years) for o/n incubation at 37 °C in complete DMEM. Cells were harvested and cytokine expression levels were evaluated in the medium using LEGENDplex Human Inflammation Panel (13-plex), according to the manufacturer instruction (740118, Biolegend).

### LPS Stimulation and Quanti Blue Assay

HEK-Blue hTLR4 and HEK-Blue hTLR2 cells were seeded into a 96-well plate (3 × 10^4^/well). After allowing overnight attachment, HEK-Blue cells were stimulated for 18 h with *E. coli* O111:B4 LPS or *P. hominis* LPS. HEK-Blue hTLR2 cells were also treated with 500 ng/mL PAM3CSK4 used as positive control of TLR2 activation. Cell supernatants were collected to assess the SEAP levels by Quanti Blue assay as indicator of NF-κB activation, following LPS stimulation, according to the manufacturer’s protocol.

### Isolation and Purification of *P. hominis* LPS

P. hominis (previously stocked in semisolid GAM broth [Gifu Anaerobic Medium, Nissui Pharmaceutical Inc., Tokyo, Japan]) was grown under strict conditions in GAM broth at 37 °C for 18 h until reaching the stationary phase. The cell pellet was collected from large-scale cultures (5 L) by centrifugation at 5000*g* for 20 min and washed twice with sterile tertiary distilled water. The pellet was freeze-dried.

The dried bacterial cells (4.322 g) were subjected to hot phenol-water extraction to isolate the LPS.^39^ Both the aqueous and phenol phases were dialyzed and further purified by enzymatic digestion with DNase and RNase (Sigma-Aldrich, Darmstadt, Germany), followed by proteinase K (Sigma-Aldrich, Darmstadt, Germany) treatments at 37 and 56 °C, respectively. To ensure the removal of any TLR2-stimulating contaminants and trace lipoproteins, an aliquot of the isolated *P. hominis* LPS, previously treated to remove nucleic acids and proteins, dialyzed, ultracentrifuged, and chromatographed, as described above, underwent several washes with a mixture of chloroform–methanol (1:2, v/v) and chloroform–methanol–water (3:2:0.25, v/v) to remove phospholipids. After the complete removal of the organic solvents, the sample was subjected to the “repurification” protocol to eliminate any residual lipoproteins.^20^ The purity and nature of the extracted material were confirmed by SDS-PAGE followed by Coomassie Brilliant Blue and silver nitrate staining, respectively.^40^ The purified LPS was analyzed using a Pierce Micro BCA protein assay (Thermo Scientific) to confirm the absence of protein contaminants.

### Chemical Analyses

The monosaccharide content was determined by analyzing the acetylated *O*-methyl glycoside derivatives obtained by treatment with HCl/MeOH (1.25 M, 85 °C, 12 h), followed by acetylation with acetic anhydride in pyridine (85 °C, 30 min). The absolute configuration of each sugar unit was determined through analysis of *O*-octylglycoside derivatives, as previously described.^41^ To determine the sugar linkage pattern, an aliquot was suspended in DMSO in the presence of NaOH and stirred for 1.5 h at room temperature. Methylation was carried out with CH_3_I, followed by hydrolysis with trifluoroacetic acid (4 M, 100 °C, 4 h), carbonyl reduction with NaBD_4_, and acetylation with pyridine and acetic anhydride. Fatty acids were extracted by treating LPS with 4 M HCl (100 °C, 4 h), followed by a treatment with 5 M NaOH (100 °C, 30 min) and extraction in chloroform, after the adjustment of the pH to reach a slight acidity. Then, fatty acids were methylated with diazomethane and analyzed via gas chromatography MS (GC–MS). All chemical analyses were conducted using gas–liquid chromatography–MS (GLC–MS) with an Agilent Technologies 6850A system equipped with a mass selective detector 5973N and a Zebron ZB-5 capillary column (Phenomenex, 30 m × 0.25 mm i.d., 0.25 μm film thickness, flow rate 1 mL/min, He as carrier gas). The temperature program for lipid analysis was 140 °C for 5 min, followed by a ramp from 140 to 280 °C at 10 °C/min, and held at 280 °C for 10 min. For sugar analysis, the program was 150 °C for 5 min, followed by a ramp from 150 to 280 °C at 3 °C/min, and held at 280 °C for 5 min.

### Isolation of LPS_OdeAc_, OS, and Lipid A Fractions

For the isolation of the LPS_OdeAc_ unit, an aliquot of LPS was dissolved in 400 μL of a 1 M aqueous solution of ammonia (NH_3_). The mixture was stirred overnight at 37 °C. After the reaction, the solution was dried under a gentle air flow, followed by freeze–drying to obtain the *O*-deacylated LPS, which was then used for subsequent structural analysis.

For the isolation of the O-antigen unit, an aliquot of LPS was subjected to mild acid hydrolysis by treating it with acetic acid at 100 °C for 4 h. This process was intended to release the repeating unit, while maintaining its acetylation. The resulting solution was extracted three times with a CHCl_3_/MeOH/H_2_O (100:30:30, v/v/v) mixture and centrifuged at 4 °C and 7000*g* for 15 min. The water-soluble fraction containing the O-antigen, acetylated repeating units was collected and lyophilized. The organic phases containing the lipid A were washed with the water phase of a freshly prepared Bligh/Dyer mixture (CHCl_3_/CH_3_OH/H_2_O, 2:2:1.8)^42^ and then freeze-dried. The purified carbohydrate and lipid A fractions were then used for subsequent structural and functional analyses.

### NMR Spectroscopy

For structural assignments of isolated polysaccharides, NMR spectra were recorded in D_2_O at 298 K at pD 7 using a cryoprobe-equipped Bruker 600 AVANCE NEO. Total correlation spectroscopy (TOCSY) experiments were performed with spinlock times of 100 ms using data sets (t1 × t2) of 4096 × 800 points. Rotating-frame Overhauser enhancement spectroscopy (ROESY) and nuclear Overhauser enhancement spectroscopy (NOESY) experiments were conducted with mixing times between 100 and 400 ms, using data sets (t1 × t2) of 4096 × 800 points. Double-quantum-filtered phase-sensitive correlation spectroscopy (DQF-COSY) experiments were performed with data sets of 4096 × 912 points. All homonuclear experiments were zero-filled in both dimensions to give a matrix of 4 K × 2 K points and were resolution-enhanced using a cosine-bell function before Fourier transformation. Coupling constants were determined by 2D phase-sensitive DQF-COSY measurements. HSQC and HMBC experiments were performed in 1H-detection mode with proton decoupling in the 13C domain, using data sets of 2048 × 512 points. HSQC was performed using sensitivity improvement in phase-sensitive mode with echo/antiecho gradient selection, including multiplicity editing during the selection step. HMBC was optimized on long-range coupling constants with a low-pass J filter to suppress one-bond correlations, using gradient pulses for selection and a 60 ms delay for the evolution of long-range correlations. HMBC spectra were optimized for 6–15 Hz coupling constants. The data matrix in all heteronuclear experiments was extended to 2048 × 1024 points using forward linear prediction extrapolation.

### MALDI MS Analysis

MALDI-TOF MS spectra were recorded on an ABSCIEX TOF/TOF 5800 Applied Biosystems mass spectrometer equipped with a Nd:laser (λ = 349 nm), with a 3 ns pulse width and a repetition rate of up to 1000 Hz. An aliquot of intact LPS was suspended in 5 mM ethylenediaminetetraacetic acid (EDTA), shaken, and briefly placed in an ultrasonic bath. The LPS was desalted with cation exchange beads (Dowex 50X, NH_4_^+^) and mixed with an equal volume of 2,5-dihydroxybenzoic acid (DHB) in 0.1% citric acid. A 1 μL aliquot of the LPS/DHB mixture was transferred to a stainless-steel MALDI plate. The MS spectrum of intact LPS was recorded in reflectron mode with negative ion polarity.^43^ The spectra were recorded in negative polarity reflectron mode. For the lipid A fraction, it was dissolved in CHCl_3_/CH_3_OH (1:1, v/v). The matrix solution was 2,4,6-trihydroxyacetophenone in CH_3_OH/0.1% trifluoroacetic acid/CH_3_CN (7:2:1, v/v) at a concentration of 75 mg/mL.^44–46^ A 0.5 μL aliquot of the sample and matrix solution was deposited on the MALDI plate and left to dry at room temperature. MS experiments were conducted with 2000 laser shots per spectrum, while MS/MS spectra accumulated 5000–7000 shots.

### ESI MS and MS/MS Analysis

LPS_DeOAc_ was dissolved in a solution composed of 50% (v/v) methanol and 50% water and directly infused by syringe pump (10 μL min^–1^, at an estimated concentration of 20 μg mL^–1^) into the source of a Waters Synapt XS mass spectrometer, equipped with a 8 kDa quadrupole operating in negative polarity, electrospray ionization (ESI), and Resolution mode. The analyses were operated with a capillary potential of 2.00 kV, a source temperature of 150 °C, a sampling cone at 60.0 V, a source offset at 0 V, a source gas (N_2_) flow at 0.0 mL min^–1^, a desolvation temperature of 600 °C, a cone gas flow at 60 L h^–1^, a desolvation gas flow at 400 L h^–1^, and a nebulizer gas pressure at 3.0 bar. MS/MS experiments were performed using ultrapure argon (SOL SpA) as collision gas, with a fixed collision energy of 23 V.

### In Silico Analyses

Molecular mechanics calculations were performed using the MM3* force field included in the MacroModel 8.0 software from the Maestro package.^47^ A dielectric constant of 80 was applied, and the disaccharide structures were explored with extended nonbonded cutoff distances (a van der Waals cutoff of 8.0% and an electrostatic cutoff of 20.0%). For each disaccharide structure, both Φ and Ψ were varied incrementally with a grid step of 18°, optimizing each (Φ, Ψ) point with 2000 P.R. conjugate gradients. MD simulations were carried out using the AMBER 18 suite of programs.^48^ Using the Leap module, the ligands were hydrated with truncated-octahedral boxes containing explicit TIP3P water molecules 15 Å away from any atom, and counterions were added to neutralize the system. After the input files were prepared, an energy minimization process was conducted to refine the initial structure. The SHAKE algorithm was applied to all hydrogen-containing bonds with a 1 fs integration step. Long-range electrostatic interactions were computed using periodic boundaries and particle-mesh Ewald summation. Isothermal and isobaric conditions were maintained during the simulation, with the temperature kept at 300 K using the Langevin thermostat (collision frequency of 1.0 ps^–1^) and the pressure maintained at 1.0 atm. Energy minimization of the solvated systems was performed with solute restraints gradually decreasing from 100 kcal/mol to no restraints. The temperature was increased from 0 to 100 K at constant volume and then from 100 to 300 K in an isobaric ensemble. The temperature was held constant at 300 K for 50 ps with progressive energy minimization and solute restraint. Once the restraints were removed, the systems advanced in an isothermal–isobaric ensemble along the production trajectory. Trajectory coordinates were sampled every picosecond to acquire 10,000 structures over the course of the dynamics. The stability of energy, pressure, temperature, and other thermodynamic parameters was monitored throughout the trajectory, with RMSD torsion values, cluster distances, and hydrogen bonds extracted using the Cpptraj module^49^ Trajectories were visualized with the VMD^50^ molecular visualization program and analyzed with the ptraj module included in AMBER18.^48^ Molecular visualization and drawing were carried out using PyMOL 2.3 ^51^ and Discovery Studio Software.^52^

### Static and Dynamic Light Scattering (SLS and DLS)

Static and DLS measurements were performed using an LSI spectrometer with a fiber-couples laser operating at 638 nm. The experiments were recorded at a constant temperature of (25.0 ± 0.1) °C using a thermostatic bath with a scattering angle θ of 90 °C. DLS measurements were carried out at a 1.0 mg/mL concentration. SLS measurements, on the other hand, were carried out over a concentration range of 1.0–0.1 mg/mL. All solutions were prepared from *P. hominis* LPS stock solution. The aqueous solution was prepared using double-distilled Milli-Q water and filters using 0.20 μm filters. Given that the size of *P. hominis* LPS is less than λ/10, we focused on concentration dependence. The average molecular weight (*M*_w_) of the polysaccharide aggregate was determined by Zimm plot analysis^53,54^

1 $$\frac{K_{c}}{R_{\theta }}=\frac{1}{M_{w}}\left[1+\frac{q^{2}\left\langle R_{g}^{2}\right\rangle }{3}+2A_{2}M_{w}c\right]$$

where c is the sample mass concentration, $K=\frac{1}{N_{A}}{\left(\frac{2\pi n}{\lambda^{2}}\frac{dn}{dc}\right)}^{2}$ a constant depending on the incident wavelength λ, the Avogadro number *N*_A_, the solvent refractive index *n*, and the refractive index variation with the polysaccharide concentration, d*n*/d*c* = 0.15. Moreover, q is the modulus of the scattering vector, and θ is the scattering angle. *M*_w_, *R*_g_, and *A*_2_ are the mass average molecular weight, the radius of gyration, and the second virial coefficient for the scattering objects. R_θ_ is the Rayleigh ratio whose expression is

2 $$R_{\theta }=\frac{\left(I_{s}-I_{0}\right)}{I_{R}}\frac{n_{0}^{2}}{n_{R}^{2}}R_{\theta ,R}$$

where *I*_s_ is the sample scattering intensity, *I*_0_ is the scattering intensity of the solvent, *I*_R_ is the scattering intensity of the reference which in our case is toluene, *n*_0_ is the refractive index of the solvent, *n*_R_ is refractive index of the reference, and *R*_θ,R_ is the Rayleigh ratio of the toluene at the incident wavelength.^55^

By plotting *K*_c_/*R*_θ_ as a function of (*hC* + sin^2^(*q*/2)), and fitting the data with extrapolation at *c* = 0, we can determine *M*_w_.

In the case of DLS experiments, the correlation function was analyzed with the CORENN algorithm, and the obtained diffusion coefficient was used to estimate the hydrodynamic radius, *R*_h_, using the Stokes–Einstein equation

3 $$R_{h}=\frac{kT}{6\pi \eta_{0}\left\langle D\right\rangle }$$

where *k* is the Boltzmann constant, *T* is the absolute temperature, and η_0_ is the solvent viscosity. This allowed us to obtain the hydrodynamic radius of the scattering object.^56–58^

Supported *P. hominis* containing bilayers deposition and NR: *P. hominis* LPS-containing bilayers were deposited on the surface of single silicon crystals using a purpose-built Langmuir–Blodgett (LB) trough (KSV-Nima, Biolin Scientific, Finland). Contrast variation was achieved by exchanging hydrogen for deuterium in the DPPC tails and in buffer solutions. LB deposition was used to create the inner leaflet of the membrane on the support, while Langmuir–Schaeffer (LS) deposition was used for the outer leaflet. For the LB deposition of the inner bilayer leaflet, d-DPPC was deposited from chloroform onto a clean, nonbuffered water subphase cooled to 10 °C containing 5 mM CaCl_2_. The phospholipid film was then compressed to a surface pressure of 35 mN m^–1^. A submerged silicon support was then lifted through the air/water interface at a speed of 4 mm/min while the surface pressure was kept constant. The LB trough was then cleaned, and an air/liquid interfacial monolayer of *P. hominis* LPS was deposited onto the cleaned surface of a nonbuffered water subphase from a solution of 2:5:8 (v/v) Phenol: Chloroform: Petroleum ether. The 2 mM CaCl_2_ subphase solution was then cooled to 10 °C. The *P. hominis* LPS monolayer was compressed to 35 mN m^–1^. For the LS deposition of the bilayer outer leaflet, the silicon support containing the LB-deposited DPPC monolayer on its surface was placed in a holder above the air/liquid interface with the angle of the crystal adjusted by using a purpose-built leveling device to make the crystal face parallel to the water surface. The silicon support (and LB film) was then dipped through the interface at a constant speed of 4 mm/min and lowered into a purpose-built sample cell in the well of the trough.

Specular NR experiments were conducted with an INTER reflectometer at the ISIS Neutron and Muon Source (Didcot, UK), using neutron wavelengths from 1 to 16 Å. Reflected intensity was measured at two glancing angles of 0.7° and 2.3° as a function of momentum transfer $Q_{z}=\frac{4\pi \sin\theta }{\lambda }$ , where λ is the wavelength and θ represents the incident angle. Custom liquid flow cells for analysis of the silicon/liquid interface were placed on a variable angle sample stage in INTER, and the inlet to the cell was connected to a liquid chromatography pump (Knauer Smartline 1000), allowing for easy exchange of the solution isotopic contrast within the solid–liquid sample cell. For each isotopic contrast, particularly we used D_2_O, H_2_O and silicon-matched water (SMW, 38% D_2_O/62% H_2_O) a total of 15 mL of 20 mM pH/D 7.2 HEPES buffer solution was pumped through the cell, ∼5 times the cell volume, at a speed of 1.5 mL/min. Collected data were analyzed using the in-house developed RasCal 2019 software (A. Hughes, ISIS). This software employs an optical matrix formalism to fit Abeles layer models to the interfacial structure. This approach describes the interface as a series of slabs, each characterized by its SLD, thickness, and roughness. Data analysis was conducted using least-squares minimization to adjust the fit parameters to reduce the differences between the model and the experimental data.

Error estimation was undertaken using RasCal’s Bayesian Error estimation routines,^59^ with the log-likelihood function described in terms of chi-squared. Marginalized posteriors were obtained using a Delayed Rejection Adaptive Metropolis algorithm,^60,61^ and the best–fit parameters taken as the distribution maxima; the uncertainties presented here are from the shortest 95%.

### Microarray Binding Assays

*P. hominis* LPS Mix1 and Mix2 and control (glyco)­proteins (fetuin, asialofetuin, ribonuclease B, and ribonuclease A, all of them from Sigma) were printed as duplicates at four different concentrations (from 1 to 0.03 mg/mL) on 16-pad nitrocellulose-coated glass slides (Grace Biolabs ONCYTE NOVA) using a noncontact arrayer (Sprint, Arrayjet Ltd.), essentially as described.^62,63^ The Cy3 fluorophore (GE Healthcare) was added at 1 μg/mL to all the solutions to enable postarray monitoring of the spots^64^ by scanning fluorescence signals upon excitation at 532 nm (green laser), using a GenePix 200-AL scanner (Axon, Molecular Devices). Commercial recombinant human innate immune lectins were as follows: Galectins 1, 3, 4, 7, 8, and 9, from ATGen; Ficolin-1, from Sino Biological Inc.; Ficolins 2 and 3, from R&D Systems Inc.; Collectin 11, Mindin and Intelectin 1, from Elabscience. Siglec-9 was produced as previously described. For testing lectin binding, the microarrays were first blocked for 1 h with 0.25% (v/v) Tween-20 in 5 mM sodium phosphate, pH 7.2, 0.2 M NaCl (PBS). After rinsing with PBS, the arrays were overlaid for 1.5 h with His-tagged lectins at 20 μg/mL in PBS buffer containing 0.1% (v/v) Tween-20 and also 4 mM β-mercaptoethanol in the case of galectins. After four washes with PBS, they were incubated for 1 h with mouse anti-His antibody precomplexed with biotinylated goat antimouse IgG antibody (Sigma, final working dilutions 1:1000 and 3:1000, respectively). For Gal-1 (not His-tagged), a 1:1000 dilution of biotinylated rabbit antihuman galectin 1 (Peprotech) was used following four further washes with PBS, and binding was detected by incubating with AlexaFluor-647 (AF647)-labeled streptavidin (Invitrogen) at 1 μg/mL in PBS, 0.1% (v/v) Tween-20, for 35 min in the dark. Finally, the slides were washed thoroughly with PBS and then with water and scanned for AF647 signals (excitation at 635 nm, red laser). Fluorescence intensities were quantified with the GenePix Pro 7 software. Goat-antilipid A antibody (Abcam, dilution 1:500, followed by biotinylated rabbit antigoat IgG, Abcam, dilution 1:1000) was also tested, giving comparable binding intensities for the two lipopolysaccharide mixes. Control assays with all the primary and secondary antibodies were performed in parallel, yielding no meaningful signals (data not shown).

## Supplementary Material





## Notes

Schematic representation of the architecture of lipopolysaccharide, sodium dodecyl sulphate-polyacrylamide gel electrophoresis, HSQC spectrum, negative-ion MALDI-TOF MS/MS spectrum, adiabatic maps, superposition of the most representative poses, torsion angle distributions, secreted cytokines evaluation, NMR assignment, observed ions and related assignment, and structural parameters (PDF)
SDS-PAGE analysis, compositional analyses, NMR spectra, MS/MS spectrum, MALDI-TOF MS analysis, and adiabatic energy maps (PDF)

## ⧫

School of Pharmacy and Institute of New Drug Development, Jeonbuk National University, Jeonju 54896, Republic of Korea

## Abbreviations

LPS: lipopolysaccharide
OS: oligosaccharide
MS: mass spectrometry
NMR: nuclear magnetic resonance
GBA: gut–brain axis
MD: molecular dynamics
DLS: dynamic light scattering
SLS: static light scattering
NR: neutron Reflectometry
