# Supplementary material for: Structure of the Lipopolysaccharide from Paenalcaligenes hominis: A Chemical Perspective on Immune Recognition
Source: JACS Au. 2025 Jun 24;5(7):3311–27. doi: 10.1021/jacsau.5c00441 (PMC12308378; doi:10.1021/jacsau.5c00441)
Supplement: Supplementary file 1 [file au5c00441_si_001.pdf]

Supporting Information

The Structure of the Lipopolysaccharide from *Paenalcaligenes hominis*: A Chemical Perspective on Immune Recognition.

Ferran Nieto-Fabregat[a], Marcello Mercogliano[a], Alessandro Cangiano[a,b], Giuseppe Vitiello[b,c], Emanuela Andretta[a], Luke A. Clifton[d], Adele Vanacore[a], Lorena Buono[e], María Asunción Campanero-Rhodes[f,g], Dolores Solís[f,g], Cristina Di Carluccio,[a,h] Giovanni Pecoraro[e], Antonio Molinaro[a,h], Giovanni Smaldone[e], Jeon-Kyung Kim[i,†], Dong-Hyun Kim[i], Luigi Paduano[a,b], Flaviana Di Lorenzo[a,h]*, Alba Silipo[a,h]*

a Department of Chemical Sciences and Task Force for Microbiome Studies, University of Naples Federico II, Via Cinthia 4, 80126, Naples, Italy. E-mail:
b CSGI, Center for Colloid and Surface Science, 50019 Sesto Fiorentino, Italy.
c Department of Chemical, Materials and Production Engineering, University of Naples Federico II, P. le Tecchio 80, 80125, Naples, Italy.
d ISIS Pulsed Neutron and Muon Source, Science and Technology Facilities Council, Rutherford Appleton Laboratory, Harwell Science and Innovation Campus, Didcot, Oxfordshire, OX11 OQX, UK.
e IRCCS SYNLAB SDN, Via G. Ferraris 144, 80146 Naples.
f Instituto de Química Física Blas Cabrera, CSIC, Serrano 119, 28006 Madrid, Spain.
g CIBER de Enfermedades Respiratorias (CIBERES), Avda Monforte de Lemos 3-5, 28029 Madrid, Spain.
h CEINGE Biotecnologie Avanzate Franco Salvatore, Via Gaetano Salvatore, 486, 80131, Naples, Italy.
i Neurobiota Research Center, College of Pharmacy, Kyung Hee University, Seoul 02447, Korea.
† School of Pharmacy and Institute of New Drug Development, Jeonbuk National University, Jeonju 54896, Republic of Korea.

Corresponding author E-mail: flaviana.dilorenzo@unina.it (FDL), silipo@unina.it (AS)

Table of Contents

Supporting Figures pag 2
Supporting Tables pag 11

Scheme S1

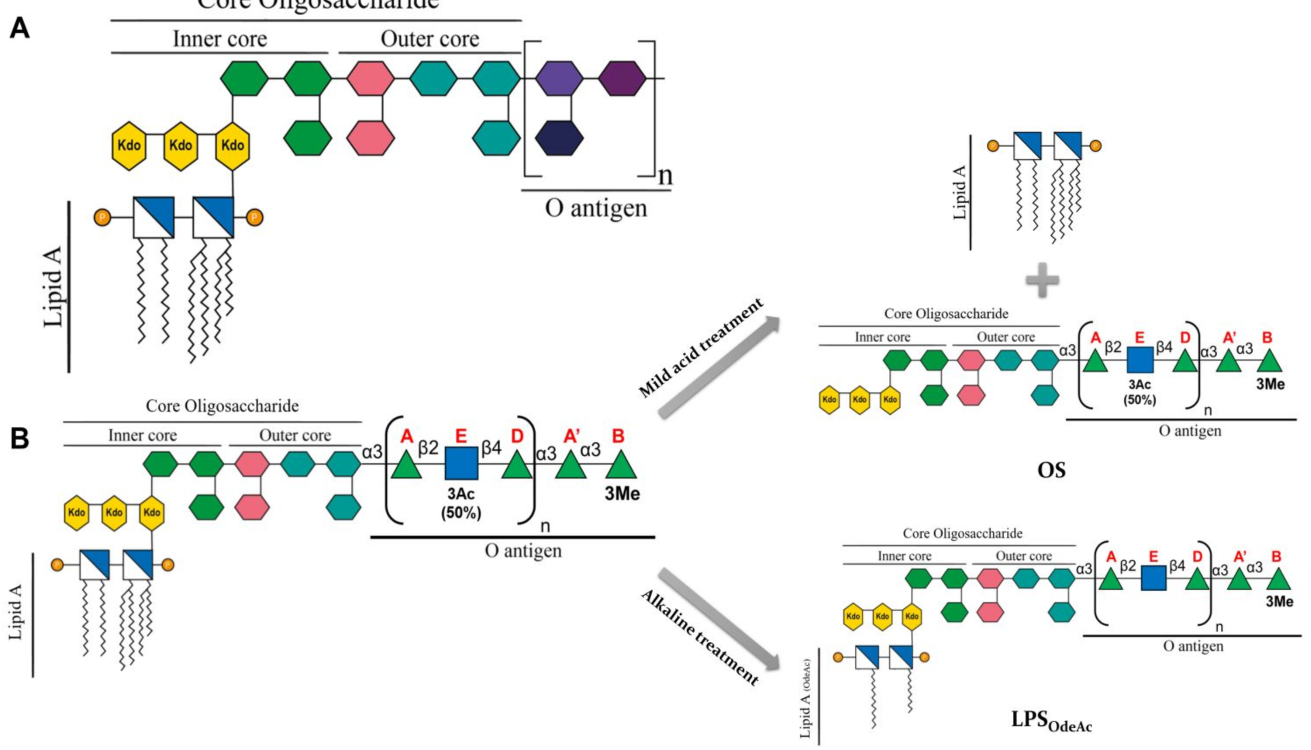

Scheme S1. A) Schematic representation of the architecture of lipopolysaccharide (LPS), composed of the lipid A domain, the core oligosaccharide (core OS) subdivided into inner and outer core regions, and the O-polysaccharide (O-antigen), which consists of repeating saccharide units. **B)** Schematic representation of the isolation strategies applied to *P. hominis* LPS and the resulting glycan structures. Following mild acid hydrolysis, selective cleavage of the Kdo–lipid A linkage yields a precipitated lipid A fraction and a supernatant containing the **OS** (O-antigen linked to the core oligosaccharide). In contrast, alkaline treatment leads to O-deacylation, generating an $\mathbf{LPS_{OdeAc}}$ preparation in which residue E (GlcN) is present in its deacetylated form. The depicted core OS and lipid A structures are schematic and do not reflect their exact composition, while the O-antigen sequence shown corresponds to the experimentally determined repeating unit and terminal cap obtained in this study.

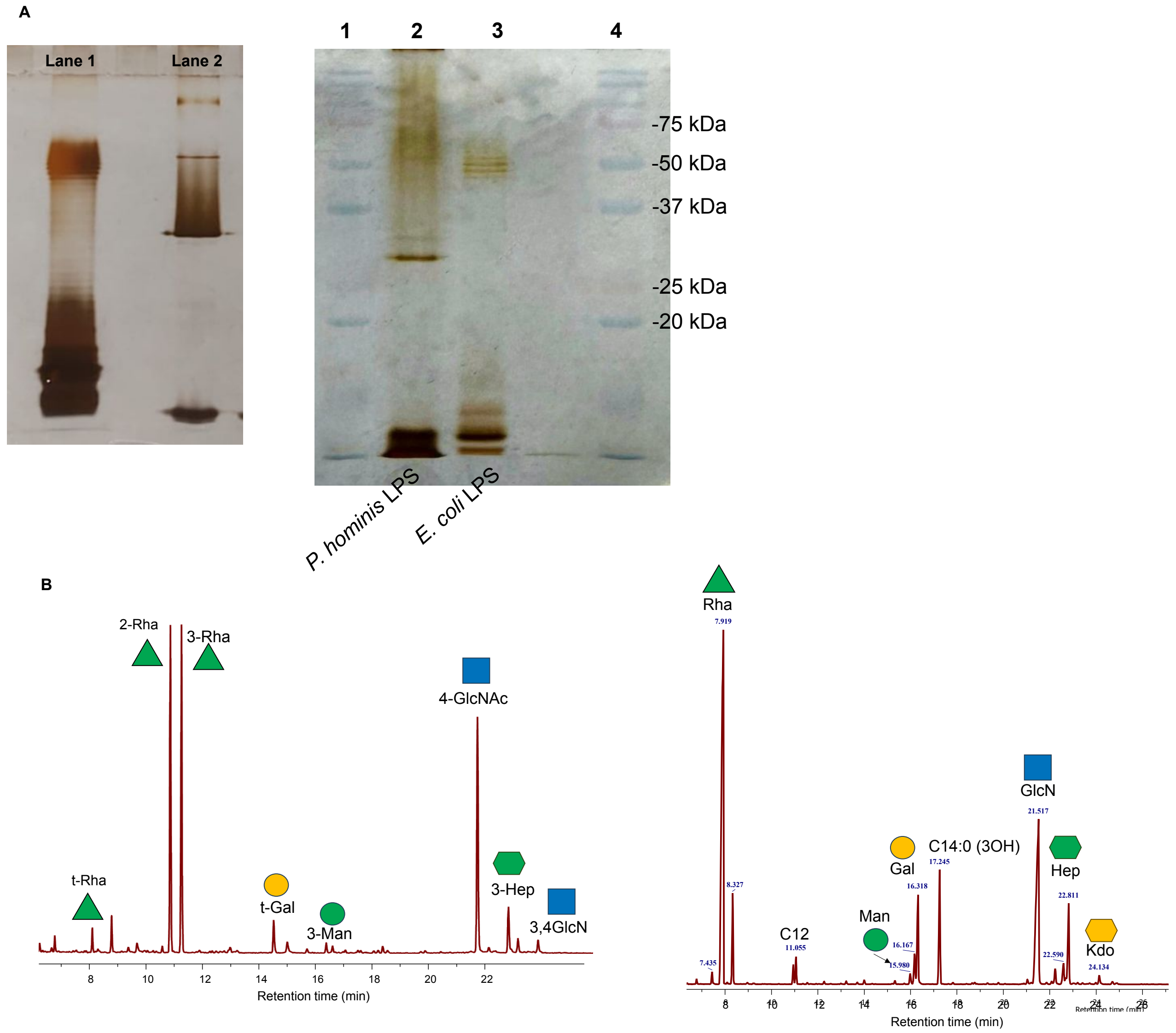

Figure S1. A) Left. Sodium dodecyl sulphate-polyacrylamide gel electrophoresis (SDS-PAGE) after silver staining of *P. hominis* LPS obtained upon enzymatic digestion (Lane 2). S-LPS from *Escherichia coli* (Lane 1) was used as a benchmark. **Right.** Silver staining of SDS-PAGE of *P. hominis* LPS. 8 µL of 1 mg/mL solution of *P. hominis* LPS (lane 2) were loaded on the gel. LPS from *E. coli* O127:B8 (8 µL, lane 3) and BLUeye Prestained Protein Ladder (2 µL, lanes 1 and 4) were used as references. **B**) GC-MS spectra: right compositional analysis and left partially methylated acetylated alditols derived from the O-antigen repeating unit of *P. hominis* LPS; signals from the core region were also highlighted. The chromatogram shows the retention times and identification of key monosaccharide derivatives colored according to Symbol Nomenclature for glycans (SNFG).

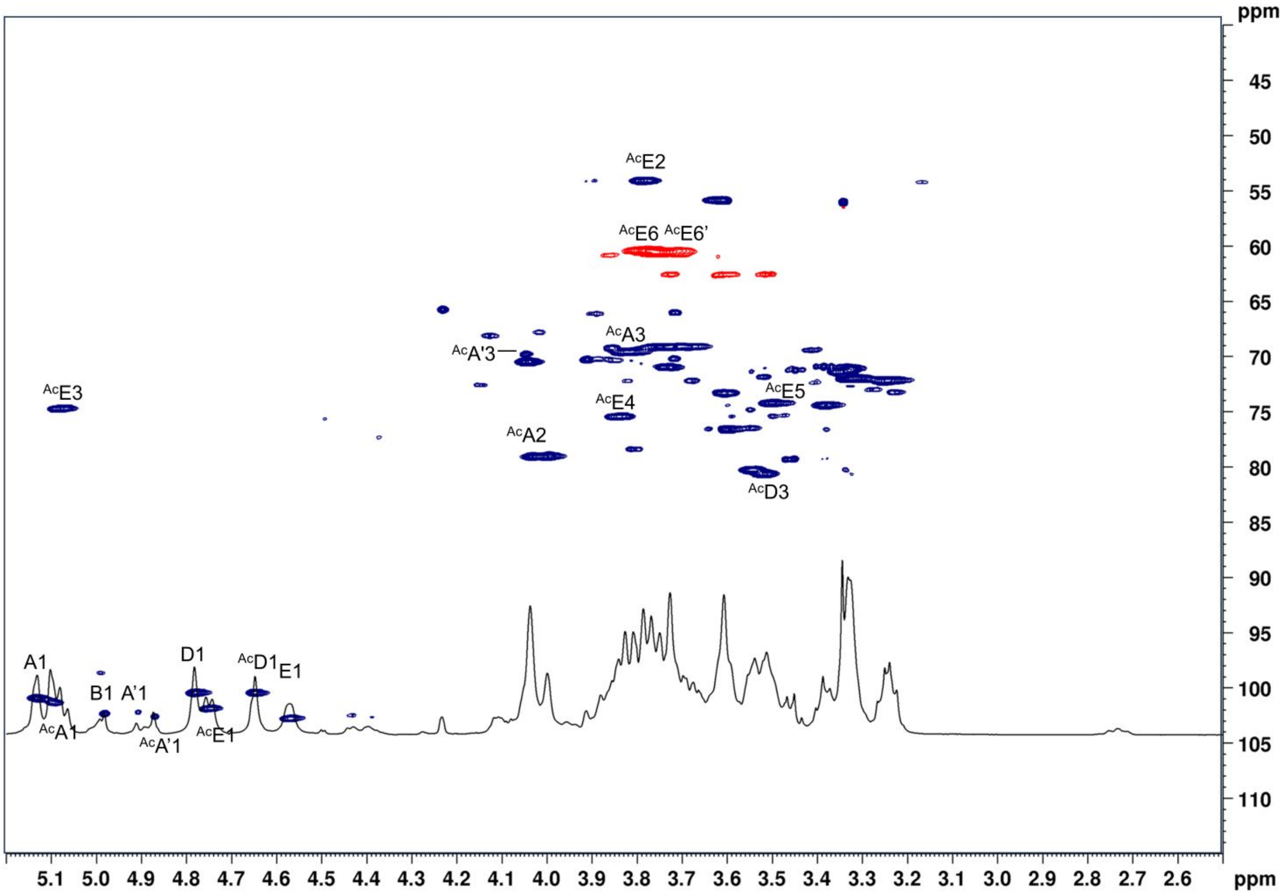

Figure S2. HSQC spectrum of the *P. hominis* O-antigen **OS** obtained from acetic hydrolysis. Due to the acetic hydrolysis, signals corresponding to the non-stoichiometrically acetylated (50%) sugar **E** are visible and thus reported in the spectrum; letters are as in Table S1.

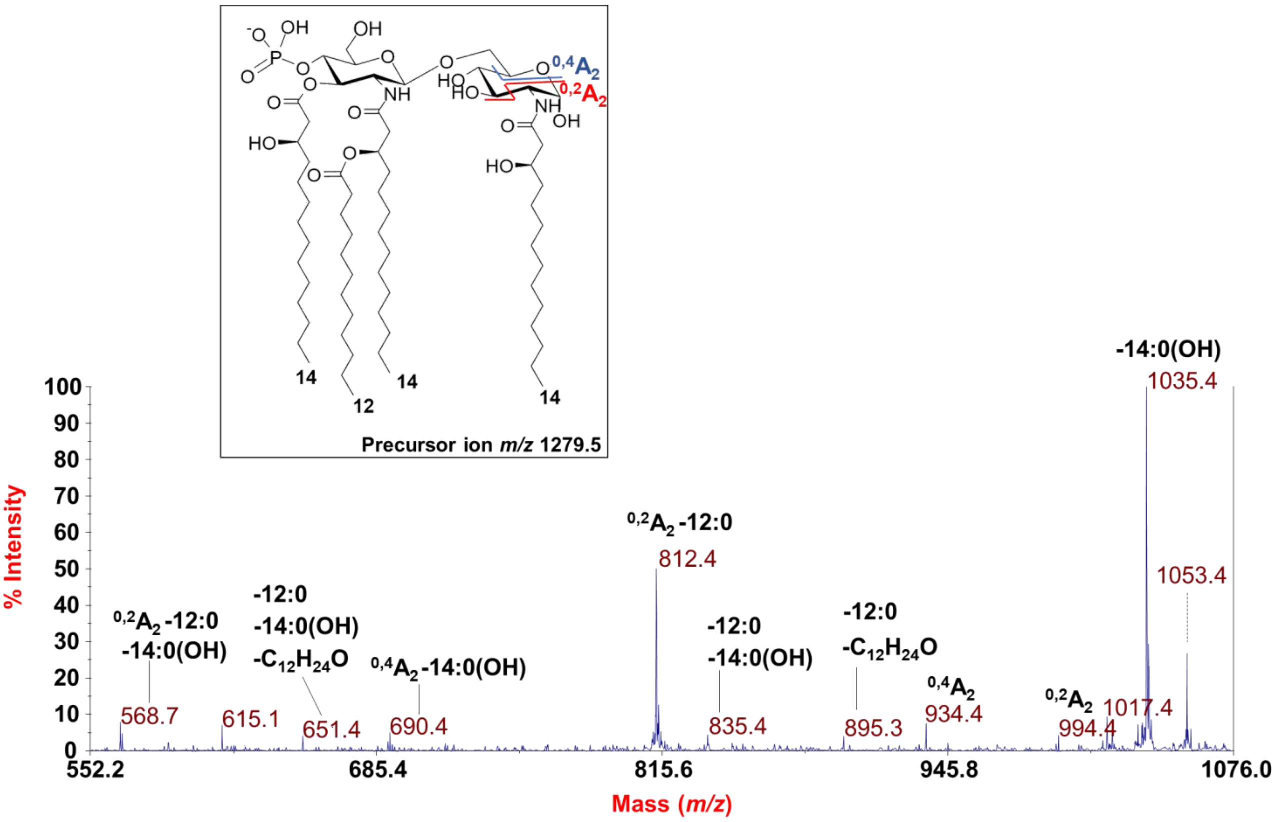

Figure S3. Negative-ion MALDI-TOF MS/MS spectrum of precursor ion at *m/z* 1279.5, relative to a *mono*-phosphorylated tetra-acylated lipid A species.

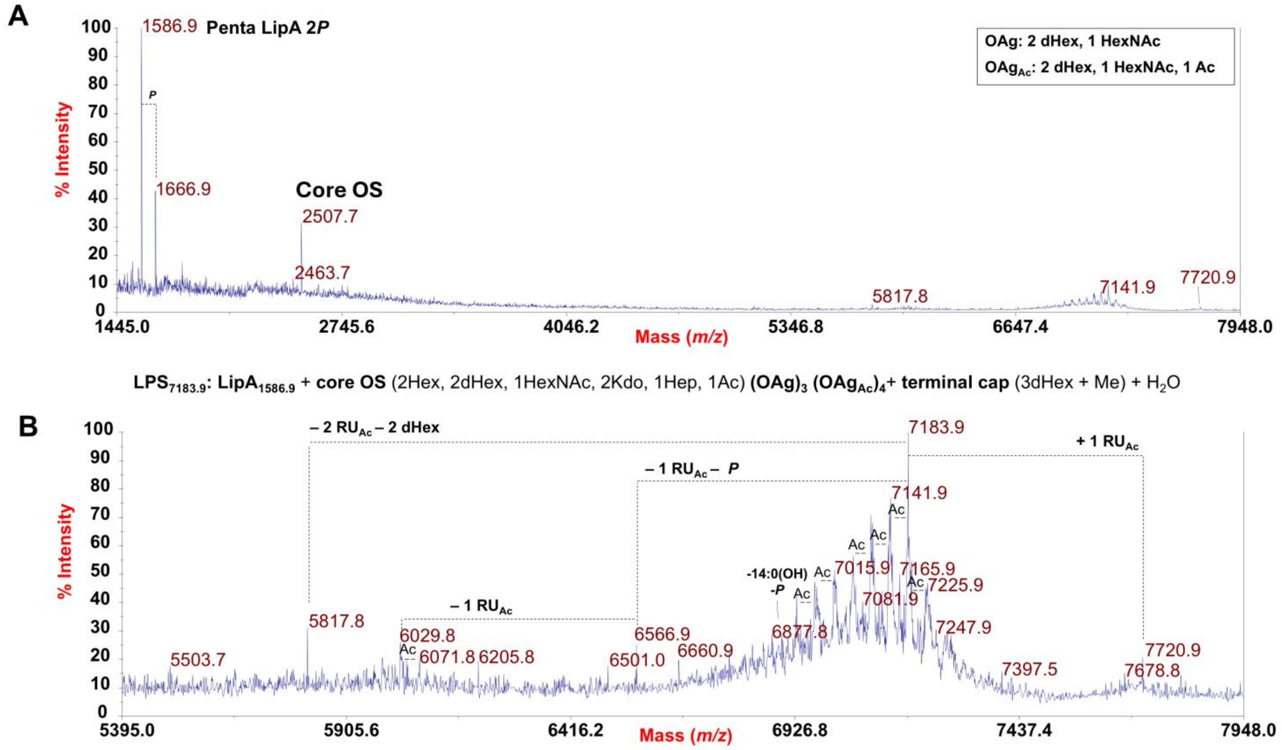

Figure S4. A) Negative-ion MALDI-TOF MS spectrum of *P. hominis* LPS, recorded in reflectron mode. **B**) Zoom of the mass range *m/z* 5395-7948 that shows peaks attributable to LPS species. OAg and OAg$_{Ac}$ stand for O-antigen repeating unit.

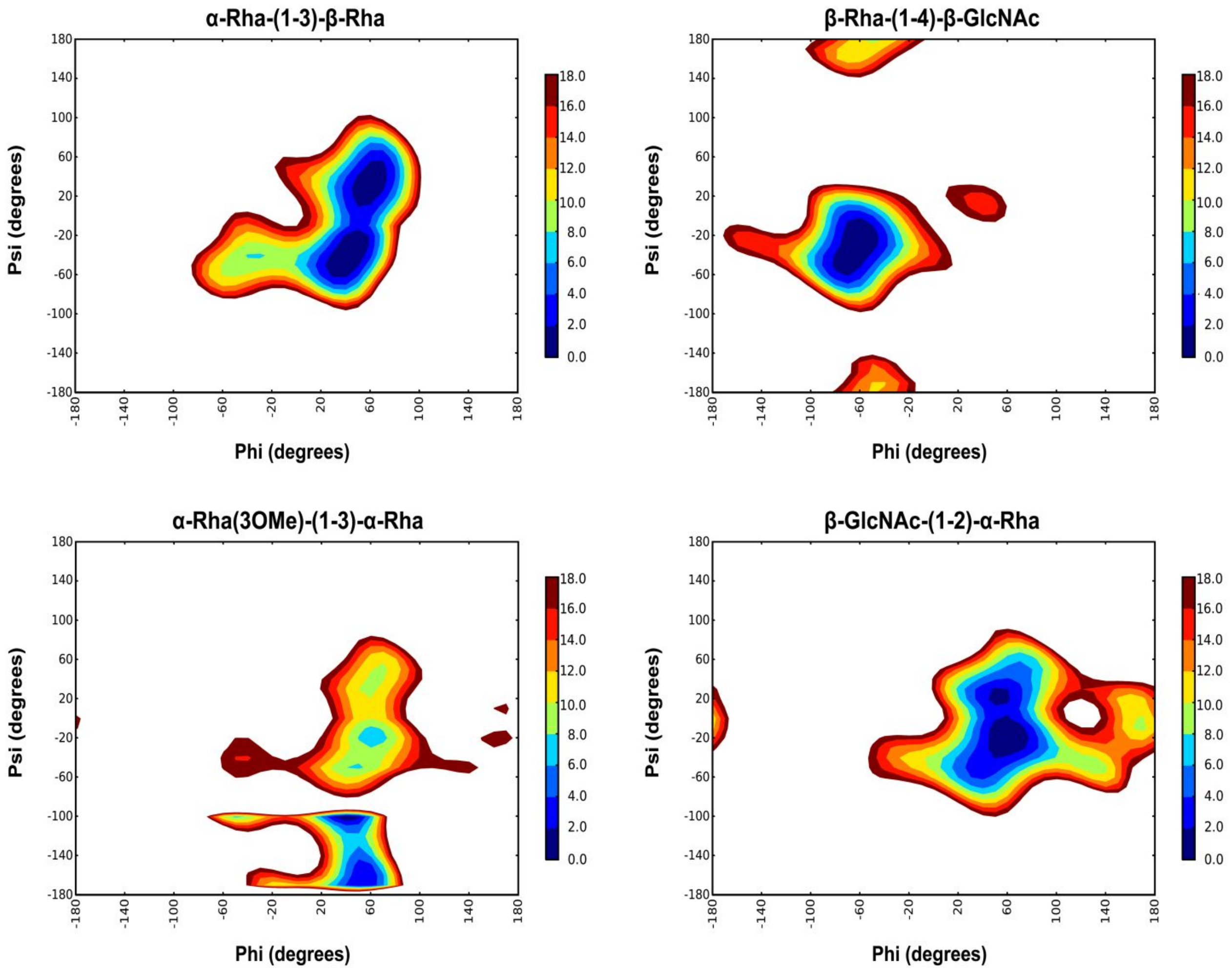

Figure S5. Adiabatic maps of Φ versus Ψ torsion angles for the glycosidic linkages in *P. hominis* LPS, calculated using molecular mechanics. The colour gradient represents the energy landscape.

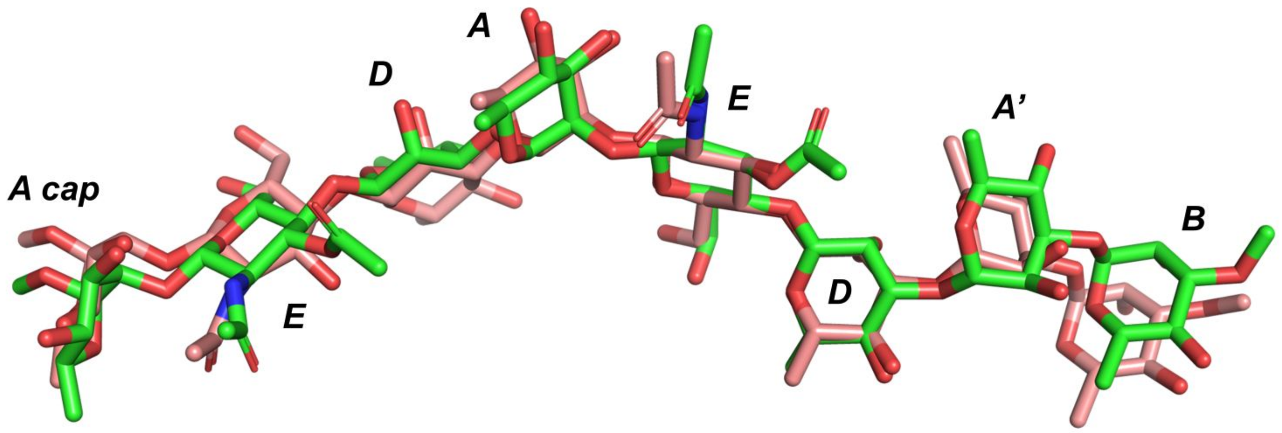

Figure S6. Superposition of the most representative poses from the MD simulations of the **OS** (green) and **LPS$_{\mathbf{OdeAc}}$** (salmon) octasaccharides

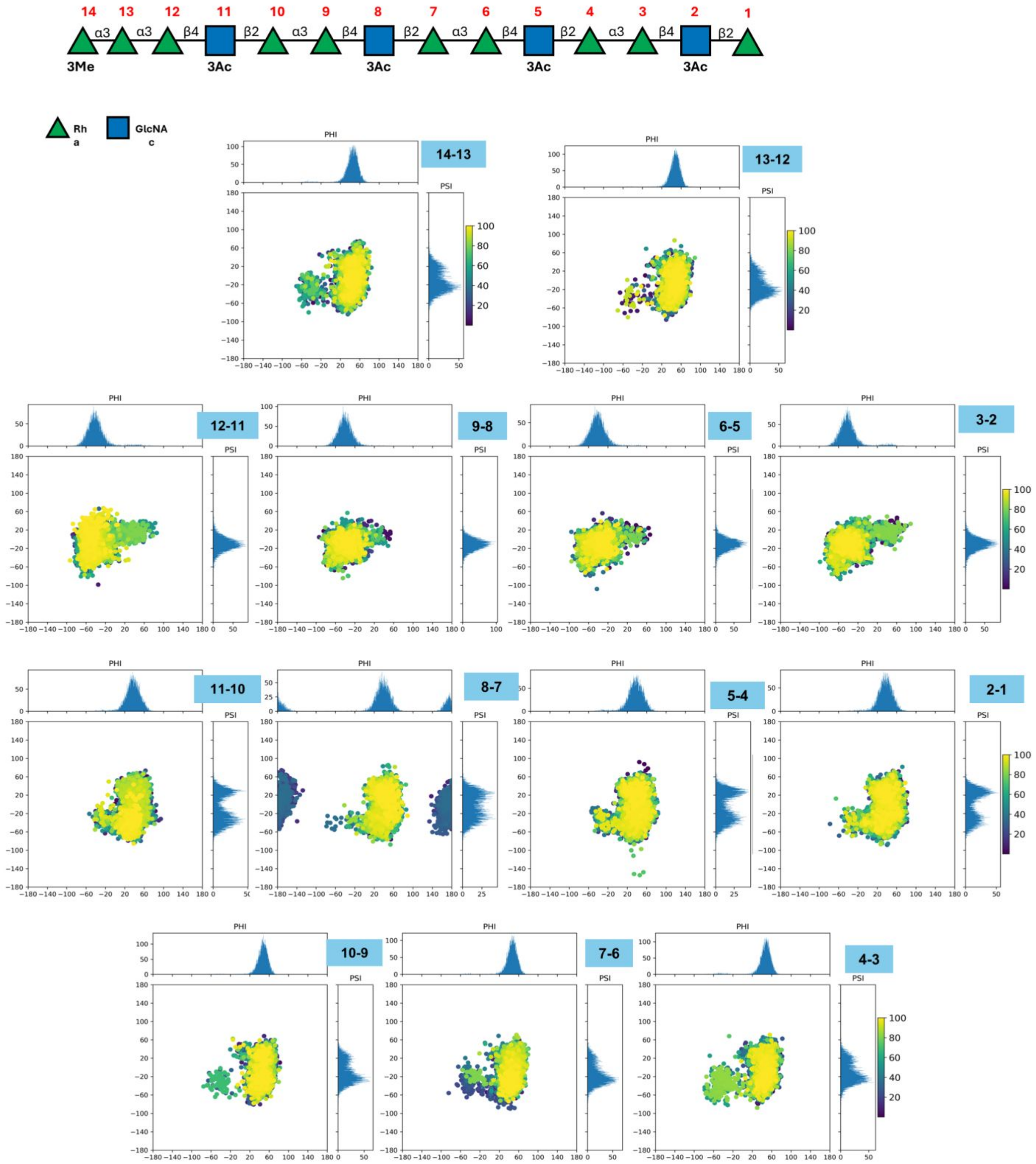

Figure S7. Torsion angle distributions around the glycosidic linkages throughout the MD simulation of *P. hominis* **OS** tetradecasaccharide. The torsion angles (Φ and Ψ) were monitored for each glycosidic bond along the entire MD simulation.

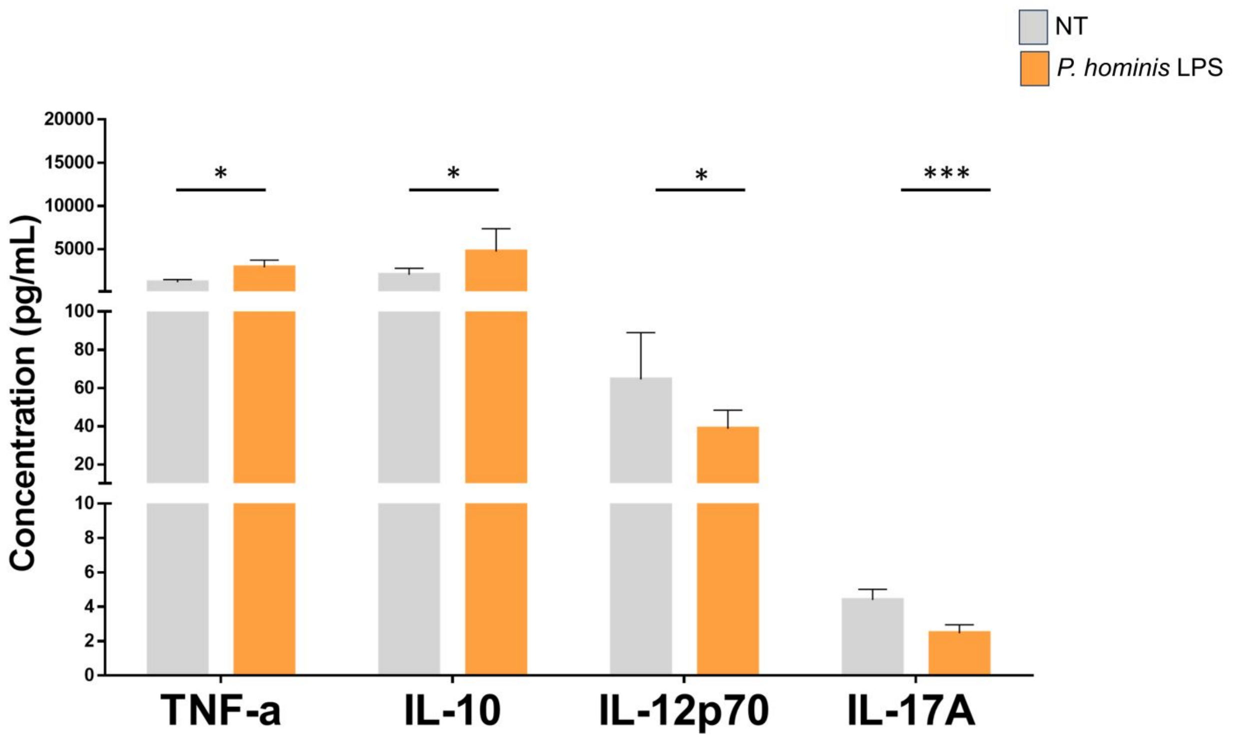

Figure S8. Secreted cytokines evaluation. Secreted TNF-α, IL-10, IL12-p70 and IL-17A expression levels (pg/ml) in 6 blood PBMCs activated with *P. hominis* LPS (orange bars) respect to the untreated PBMCs (grey bars). *= *p*-value<0,05. ***= *p*-value< 0,001. Paired *t*-test.

Table S1 NMR assignment of *P. hominis* LPS O-antigen. The ^{1}H and ^{13}C NMR chemical shifts for each sugar residue were reported on the first and second rows, respectively. Here the structure of Rha and GlcNAc residues. $^{1}J_{CH}$ values for the definition of the anomeric configuration were also reported

**LPS_{OdeAc}**						
	**1**	**2**	**3**	**4**	**5**	**6**
**A**	5.15	4.02	3.84	3.27	3.73	1.16
2-α-Rha	100.9	79.1	69.7	72.2	69	16.9
	$^{1}J_{CH}$= 172.2 Hz					
**B**	5.01	4.26	3.49	3.41	3.79	1.23
t-α-Rha(3OMe)	102.5	65.8	79.3	70.9	69	16.9
	$^{1}J_{CH}$= 171.2 Hz	^{1}H and ^{13}C resonances of O-Methyl group				
			3.36/56.0			
**C**	102.3	69.7	78.4	71.3	69	16.9
3-α-Rha	4.93	4.08	3.85	3.48	3.8	1.23
	$^{1}J_{CH}$= 173.4 Hz					
**D**	4.81	4.06	3.56	3.36	3.35	1.23
3-β-Rha	100.5	70.5	80.4	71.3	72.3	16.9
	$^{1}J_{CH}$= 163.0 Hz					
**E**	4.6	3.65	3.41	3.62	3.63	3.81 3.75
4-β-GlcNAc	102.7	55.9	74.4	76.7	73.5	60.6
	$^{1}J_{CH}$= 166.1 Hz					
	^{1}H and ^{13}C resonances of N-Acetyl group					
		1.97/22.2				
**O-antigen**						
**E**	4.76	3.79	5.08	3.85	3.49	3.75/3.69
4-β-GlcNAc(3OAc)	101.9	54	74.6	75.4	75.5	61
	$^{1}J_{CH}$= 165.6 Hz					
	^{1}H and ^{13}C resonances of N-Acetyl and O-Methyl groups					

		1.89/21.98	2.05/20.29			
**E’**	4.57	3.63	3.38	3.59	362	3.77
4-β-GlcNAc	102.73	55.8	74.4	76.58	73.3	60.44
	$^{1}J_{CH}$= 166.0 Hz					
	^{1}H and ^{13}C resonances of N-Acetyl group					
		1.95/22.21				

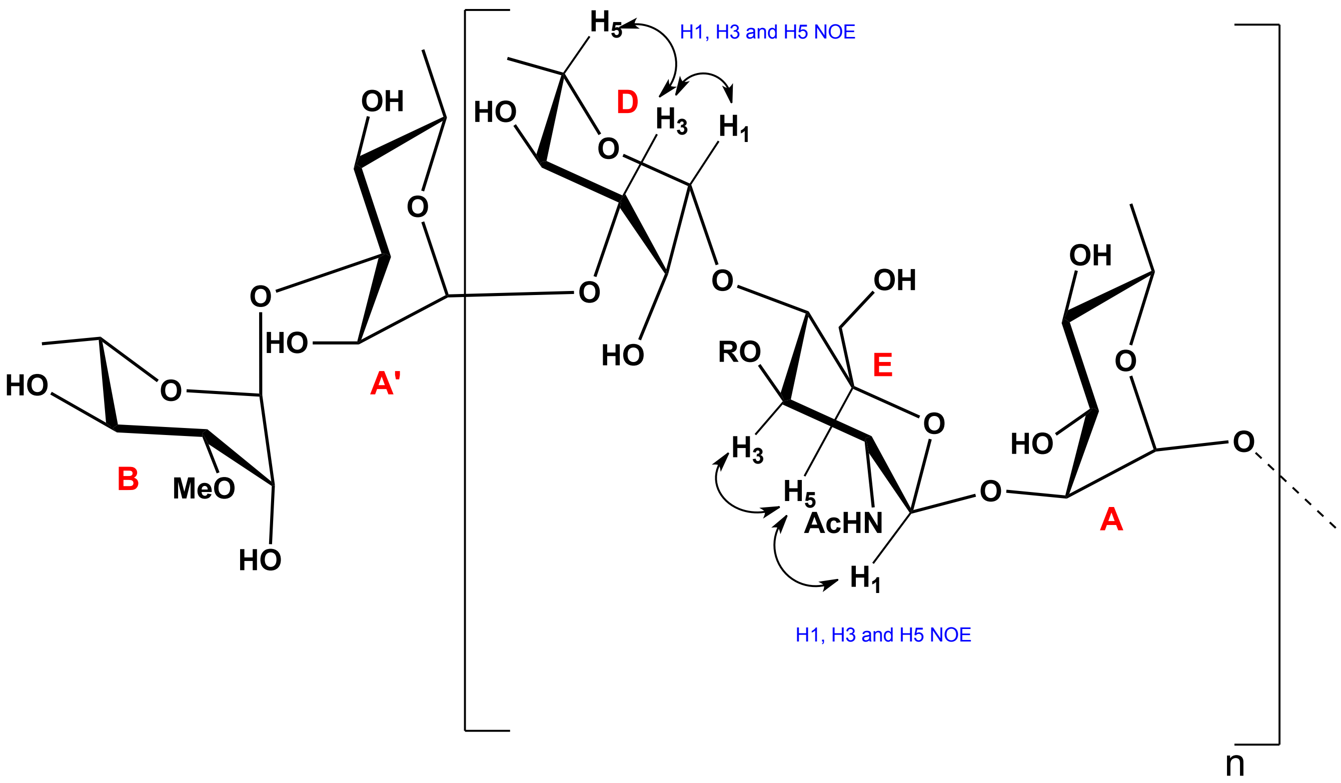

Table S2. Observed Ions and related assignment derived from ESI-MS of *O*-deacylated *P. hominis* LPS

Observed ion peaks (*m/z*)	Proposed structure	Proposed composition	Interpretation of the Ion
834.94	Core OS + $(OAg_{Ac})_2$ + HexNAc	$(Hex)_2$, Hep, Kdo, $(dHex)_6$, $(HexNAc)_4$, $(Ac)_2$	$[M\text{-}3H]^{3-}$
871.51	LipA1 - *P*	$(HexN)_2$, $[14{:}0(3\text{-}OH)]_2$	$[M\text{-}H]^{-}$
951.47	LipA1	$(HexN)_2$, $[14{:}0(3\text{-}OH)]_2$, P	$[M\text{-}H]^{-}$
1053.68	LipA2 - *P*	$(HexN)_2$, $[14{:}0(3\text{-}OH)]_2$, (12:0)	$[M\text{-}H]^{-}$
1133.60	LipA2	$(HexN)_2$, $[14{:}0(3\text{-}OH)]_2$, (12:0), P	$[M\text{-}H]^{-}$
1163.78	LPS3 – (OAg) – $(OAg_{Ac})_2$	$(Hex)_2$, Hep, $(Kdo)_2$, $(dHex)_{14}$, $(HexNAc)_6$, $(Ac)_2$, dHexOMe, $(HexN)_2$, $[14{:}0(3\text{-}OH)]_3$, (12:0), P	$[M\text{-}5H]^{5-}$
1230.42	Core OS (B-type ion)	$(Hex)_2$, HexNAc, $(dHex)_2$, Hep, Kdo	$[M\text{-}H]^{-}$
1272.43	Core OS_{Ac} (B-type ion)	$(Hex)_2$, HexNAc, $(dHex)_2$, Hep, Kdo, Ac	$[M\text{-}H]^{-}$
1359.73	LipA3	$(HexN)_2$, $[14{:}0(3\text{-}OH)]_3$, (12:0), *P*	$[M\text{-}H]^{-}$
1387.58	LPS - Ac	$(Hex)_2$, Hep, $(Kdo)_2$, $(dHex)_{20}$, $(HexNAc)_9$, $(Ac)_3$, dHexOMe, $(HexN)_2$, $[14{:}0(3\text{-}OH)]_2$, P	$[M\text{-}5H]^{5-}$
1395.94	LPS	$(Hex)_2$, Hep, $(Kdo)_2$, $(dHex)_{20}$, $(HexNAc)_9$, $(Ac)_4$, dHexOMe, $(HexN)_2$, $[14{:}0(3\text{-}OH)]_2$, *P*	$[M\text{-}5H]^{5-}$
1586.44	LPS + (OAg) + (OAg_{Ac}) - *P*	$(Hex)_2$, Hep, $(Kdo)_2$, $(dHex)_{24}$, $(HexNAc)_{11}$, $(Ac)_5$, dHexOMe, $(HexN)_2$, $[14{:}0(3\text{-}OH)]_2$	$[M\text{-}5H]^{5-}$
1622.88	LPS2 + (OAg) + (OAg_{Ac}) - *P*	$(Hex)_2$, Hep, $(Kdo)_2$, $(dHex)_{24}$, $(HexNAc)_{11}$, $(Ac)_5$, dHexOMe, $(HexN)_2$, $[14{:}0(3\text{-}OH)]_2$, (12:0)	$[M\text{-}5H]^{5-}$
1668.12	LPS3 + (OAg) + (OAg_{Ac}) - *P*	$(Hex)_2$, Hep, $(Kdo)_2$, $(dHex)_{24}$, $(HexNAc)_{11}$, $(Ac)_5$, dHexOMe, $(HexN)_2$, $[14{:}0(3\text{-}OH)]_3$, (12:0)	$[M\text{-}5H]^{5-}$
1745.18	LPS	$(Hex)_2$, Hep, $(Kdo)_2$, $(dHex)_{20}$, $(HexNAc)_9$, $(Ac)_4$, dHexOMe, $(HexN)_2$, $[14{:}0(3\text{-}OH)]_2$, *P*	$[M\text{-}4H]^{4-}$

Table S3 Structural parameters obtained for an asymmetric d-DPPC (Inner Leaflet)/*P. hominis* LPS (Outer Leaflet) bilayer deposited on a silicon surface

**Layer**	**Thickness [Å]**	**SLD x 10^{-6} [$Å^{-2}$]**	**Roughness [Å]**	**Hydration %**
Silicon Oxide	18.15	3.18	2.5	22.5
Inner Headgroups	6.00	2.1	-	30
Inner Tails	18.3	-	-	-
Outer Tails	12.0	-	-	-
Whole lipid bilayer	-	-0.37	4.1	16.4
Core Region	19.95	4.2 (D_2O)<br>2.01 (H_2O)<br>2.84 (SiMW)	-	26.16
O-Antigen region	90	2.25	41.6	81.3
