# Supplementary material for: Structure of the Lipopolysaccharide from Paenalcaligenes hominis: A Chemical Perspective on Immune Recognition
Source: JACS Au. 2025 Jun 24;5(7):3311–27. doi: 10.1021/jacsau.5c00441 (PMC12308378; doi:10.1021/jacsau.5c00441)
Supplement: Supplementary file 2 [file au5c00441_si_002.pdf]

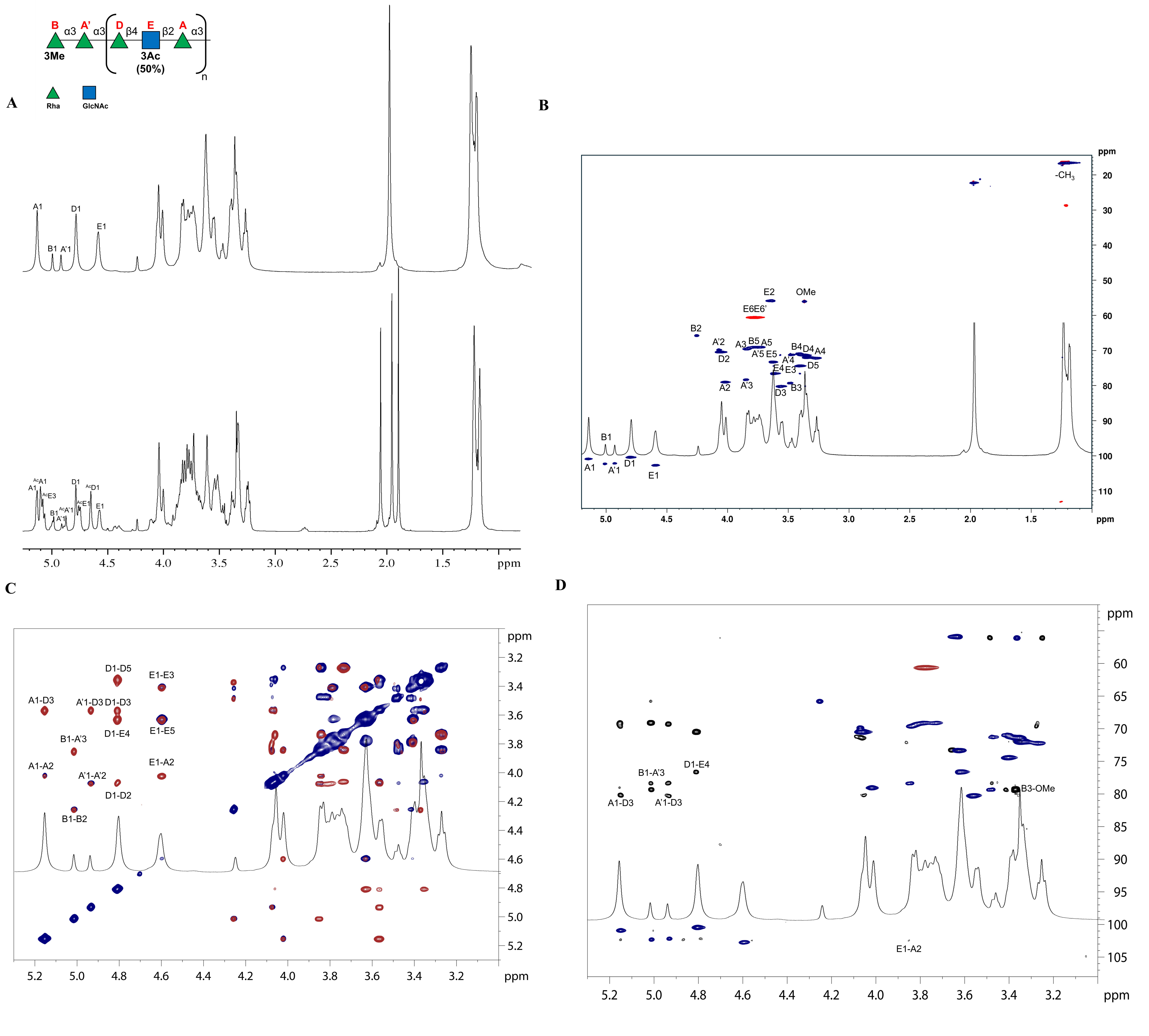
B
A'
D
E
A
α3
α3
β4
β2
α3
3Me
3Ac
(50%)
n
Rha
GlcNAc
A
A1
B1
A'1
D1
E1
A1
AcA1
AcE3
B1
AcA'1
A'1
D1
AcE1
AcD1
E1
5.0
4.5
4.0
3.5
3.0
2.5
2.0
1.5
ppm
B
-CH3
E2
OMe
E6E6'
B2
A'2
A3
B5
A5
A'5
E5
B4
D4
A4
D2
A'4
E4
E3
D5
A2
A'3
D3
B3
B1
A1
A'1
D1
E1
C
D1-D5
E1-E3
A1-D3
A'1-D3
D1-D3
D1-E4
E1-E5
B1-A'3
A1-A2
E1-A2
A'1-A2
D1-D2
B1-B2
D
B1-A'3
D1-E4
B3-OMe
A1-D3
A'1-D3
E1-A2

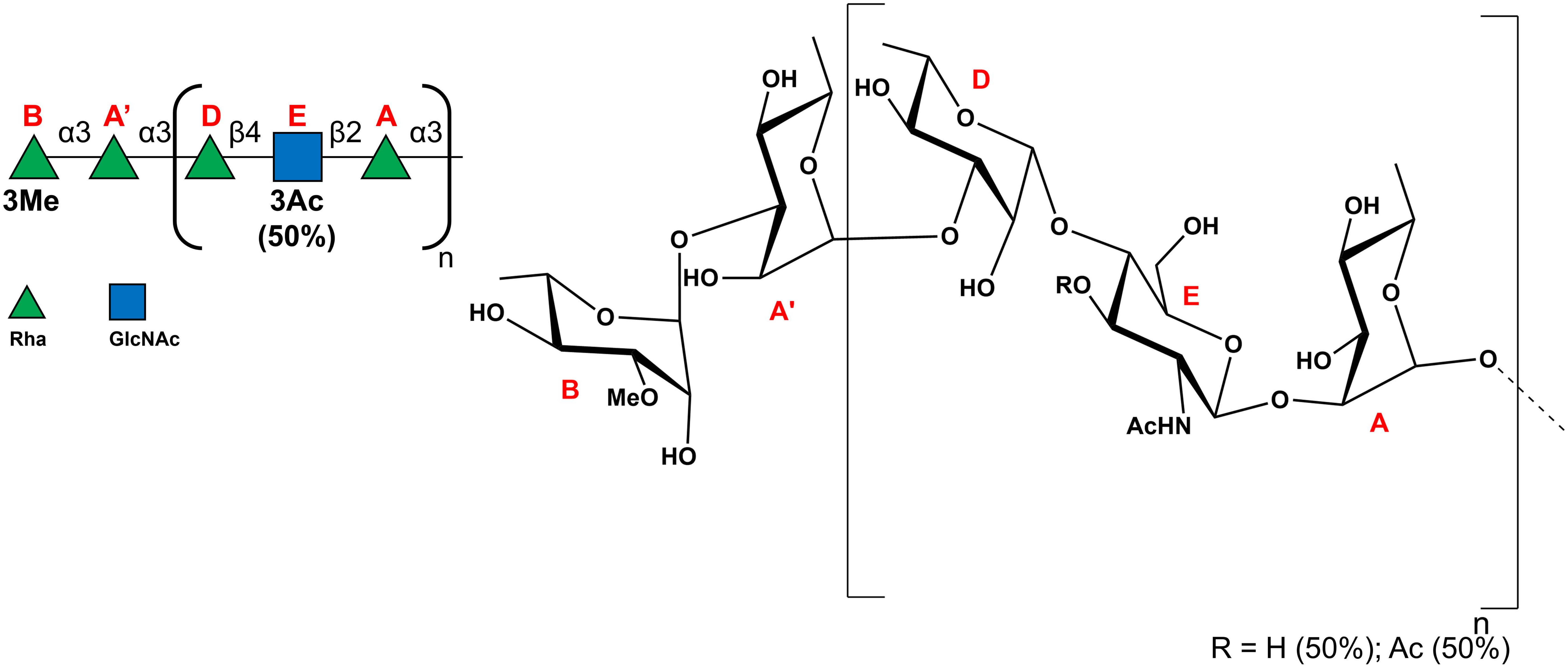

B
α3
A'
α3
D
β4
E
β2
A
α3
3Me
3Ac
(50%)
n
Rha
GlcNAc
R = H (50%); Ac (50%)

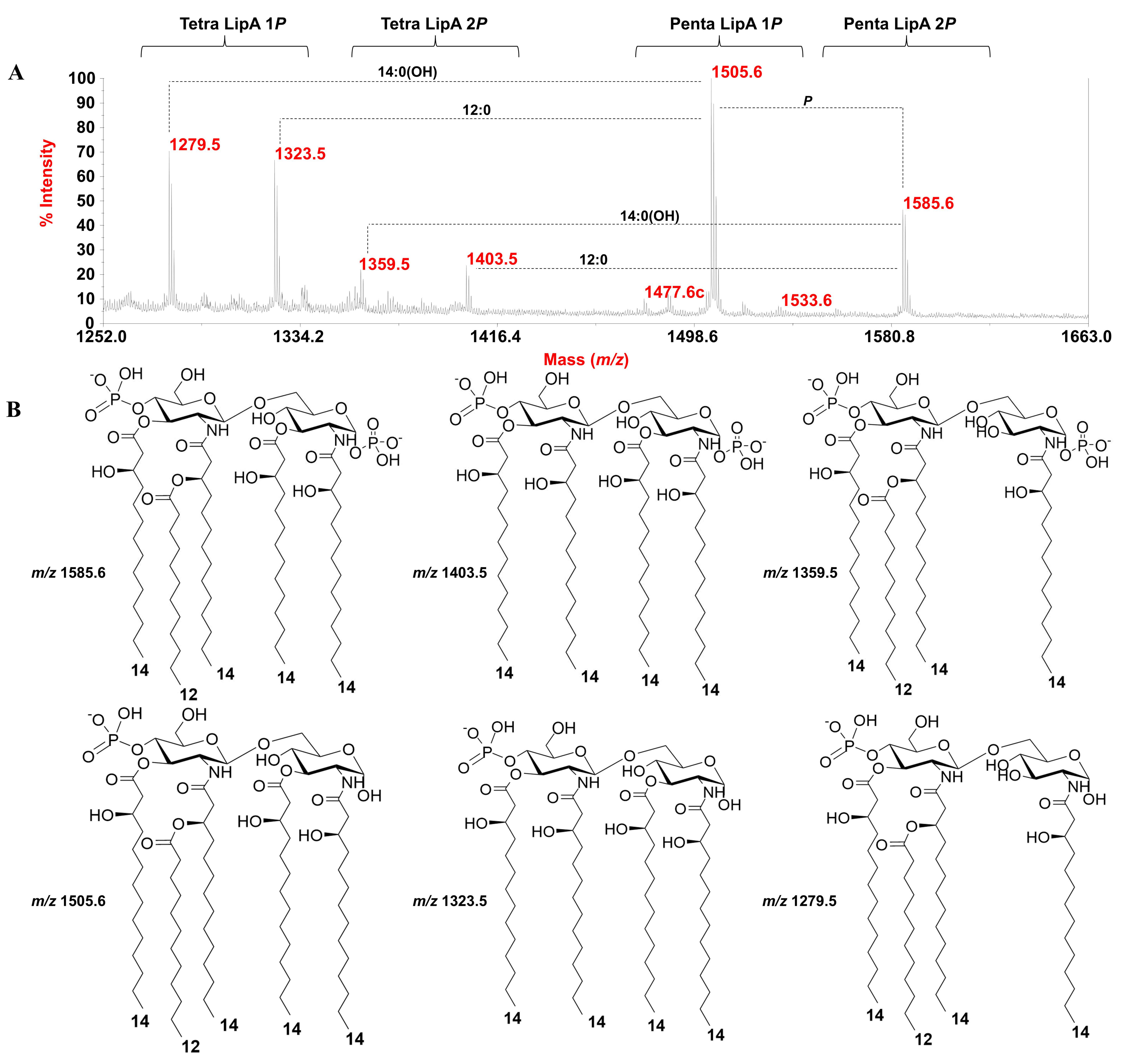

Tetra LipA 1P
Tetra LipA 2P
Penta LipA 1P
Penta LipA 2P
A
% Intensity
Mass (m/z)
14:0(OH)
12:0
P
14:0(OH)
12:0
1505.6
1279.5
1323.5
1585.6
1359.5
1403.5
1477.6c
1533.6
B
m/z 1585.6
m/z 1403.5
m/z 1359.5
m/z 1505.6
m/z 1323.5
m/z 1279.5

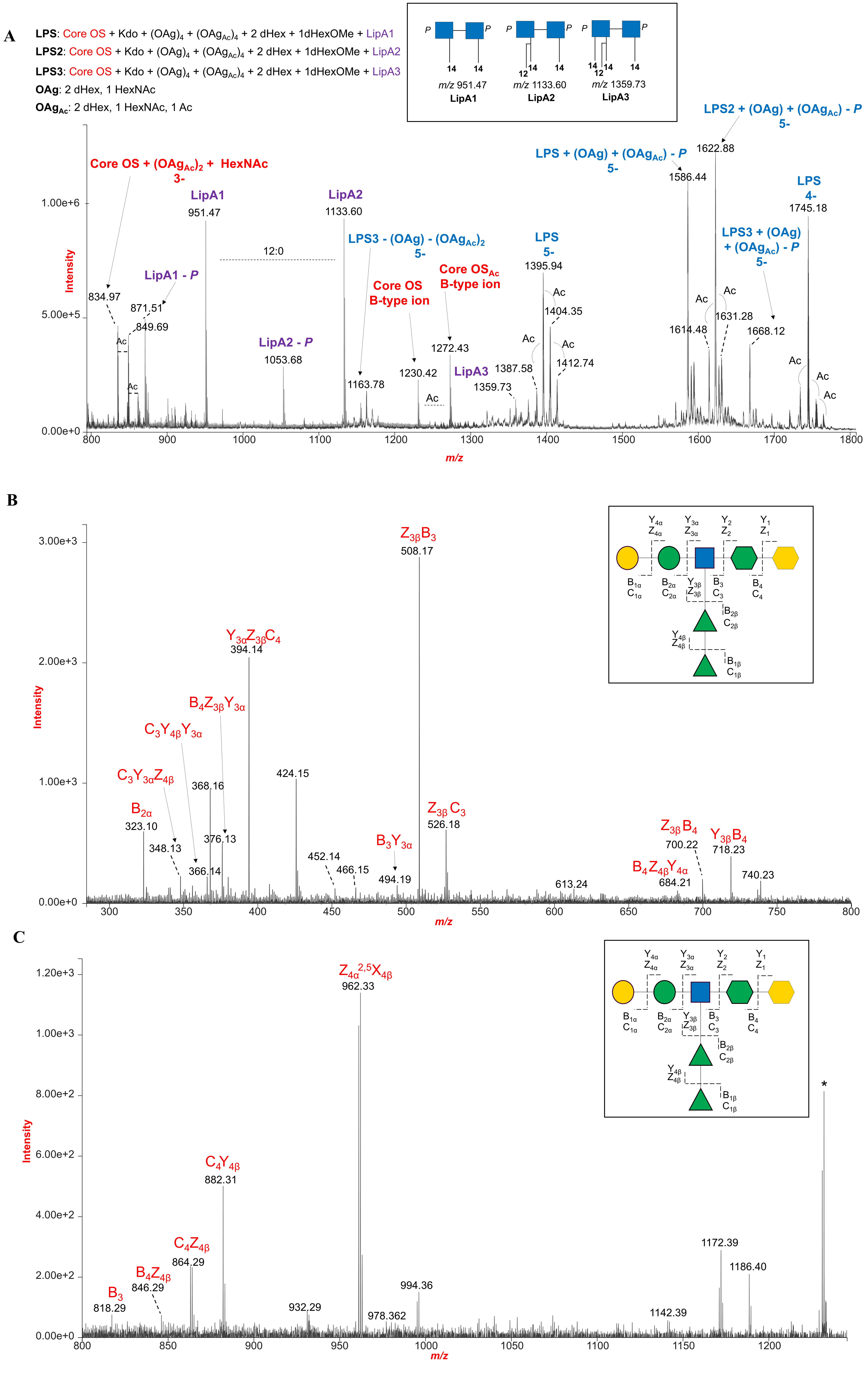
A
LPS: Core OS + Kdo + (OAg)4 + (OAgAc)4 + 2 dHex + 1dHexOMe + LipA1
LPS2: Core OS + Kdo + (OAg)4 + (OAgAc)4 + 2 dHex + 1dHexOMe + LipA2
LPS3: Core OS + Kdo + (OAg)4 + (OAgAc)4 + 2 dHex + 1dHexOMe + LipA3
OAg: 2 dHex, 1 HexNAc
OAgAc: 2 dHex, 1 HexNAc, 1 Ac
m/z 951.47 LipA1
m/z 1133.60 LipA2
m/z 1359.73 LipA3
Core OS + (OAgAc)2 + HexNAc 3-
LipA1 951.47
LipA1 - P
LipA2 1133.60
LipA2 - P 1053.68
12:0
LPS3 - (OAg) - (OAgAc)2 5-
Core OS B-type ion
Core OSAc B-type ion
LipA3 1359.73
LPS 5- 1395.94
LPS + (OAg) + (OAgAc) - P 5-
LPS2 + (OAg) + (OAgAc) - P 5-
LPS3 + (OAg) + (OAgAc) - P 5-
LPS 4- 1745.18
Intensity
m/z
B
Z3βB3 508.17
Y3αZ3βC4 394.14
B4Z3βY3α
C3Y4βY3α
C3Y3αZ4β
B2α 323.10
Z3βC3 526.18
B3Y3α 494.19
Z3βB4 700.22
Y3βB4 718.23
B4Z4βY4α 684.21
C
Z4α2,5X4β 962.33
C4Y4β 882.31
C4Z4β 864.29
B4Z4β 846.29
B3 818.29
*

Figure 5

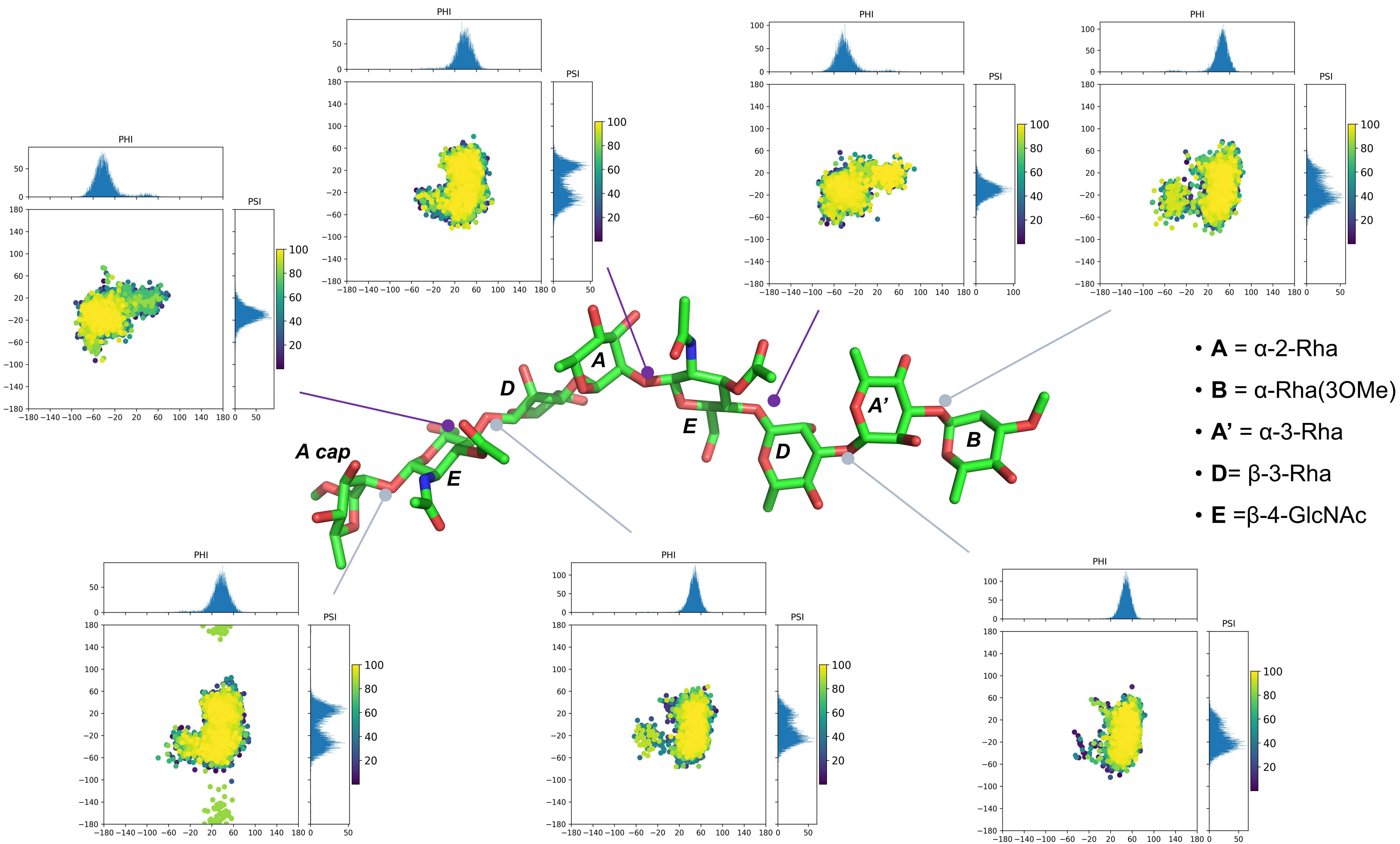
PHI
PSI
A cap
A
D
E
A'
B
• A = α-2-Rha
• B = α-Rha(3OMe)
• A' = α-3-Rha
• D= β-3-Rha
• E =β-4-GlcNAc

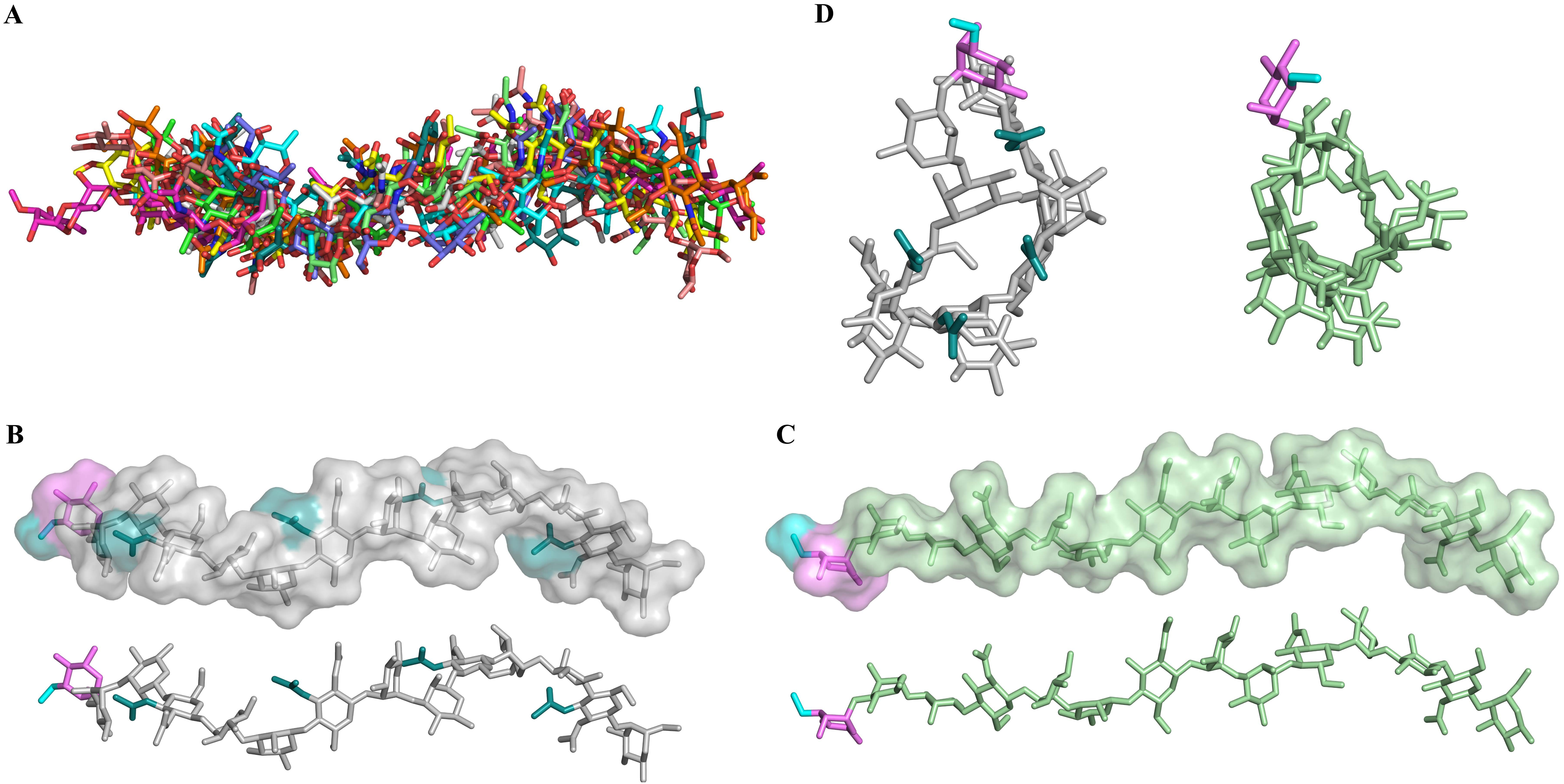
A
D
B
C

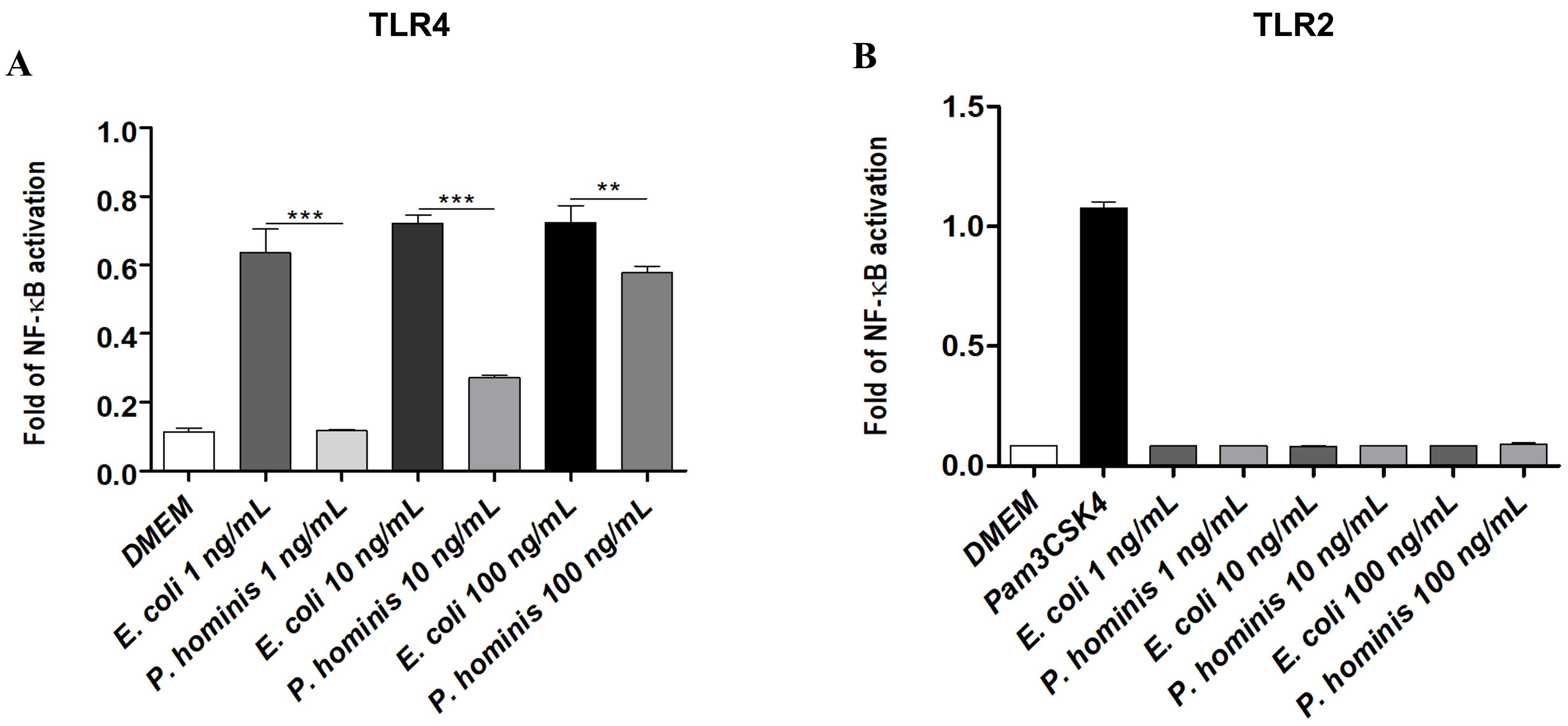

A
TLR4
Fold of NF-κB activation
1.0
0.8
0.6
0.4
0.2
0.0
***
***
**
DMEM
E. coli 1 ng/mL
P. hominis 1 ng/mL
E. coli 10 ng/mL
P. hominis 10 ng/mL
E. coli 100 ng/mL
P. hominis 100 ng/mL
B
TLR2
Fold of NF-κB activation
1.5
1.0
0.5
0.0
DMEM
Pam3CSK4
E. coli 1 ng/mL
P. hominis 1 ng/mL
E. coli 10 ng/mL
P. hominis 10 ng/mL
E. coli 100 ng/mL
P. hominis 100 ng/mL

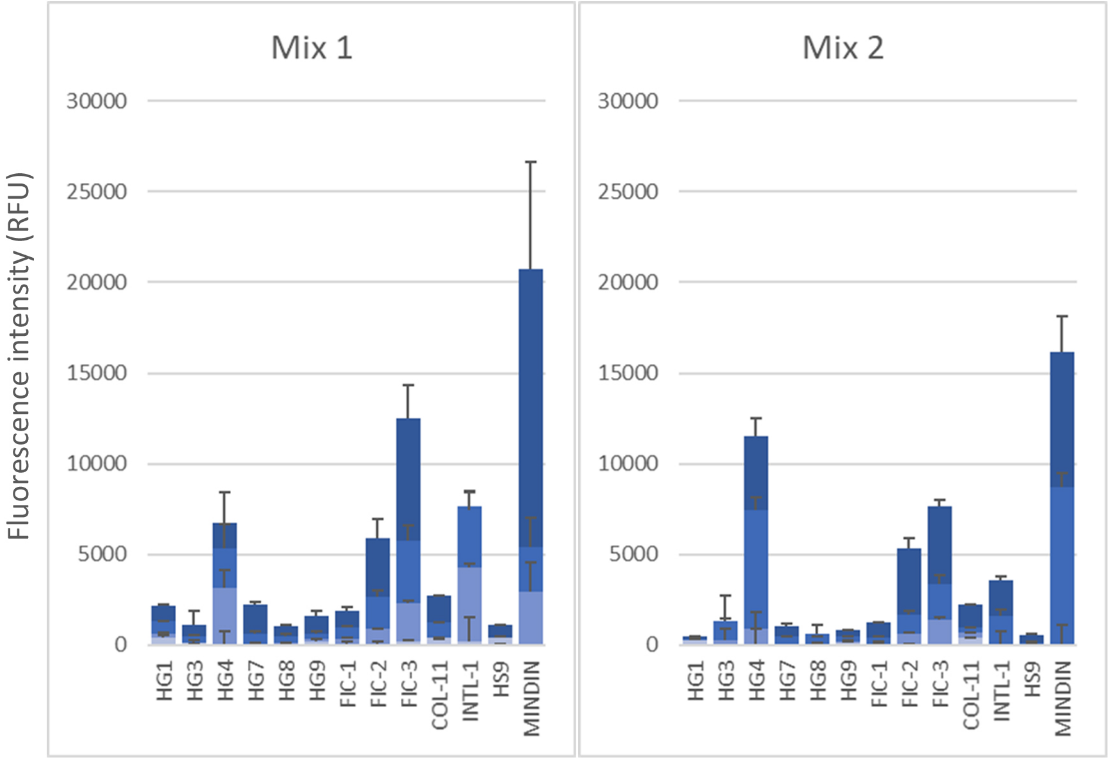
Mix 1
Mix 2
Fluorescence intensity (RFU)
30000
25000
20000
15000
10000
5000
0
HG1
HG3
HG4
HG7
HG8
HG9
FIC-1
FIC-2
FIC-3
COL-11
INTL-1
HS9
MINDIN

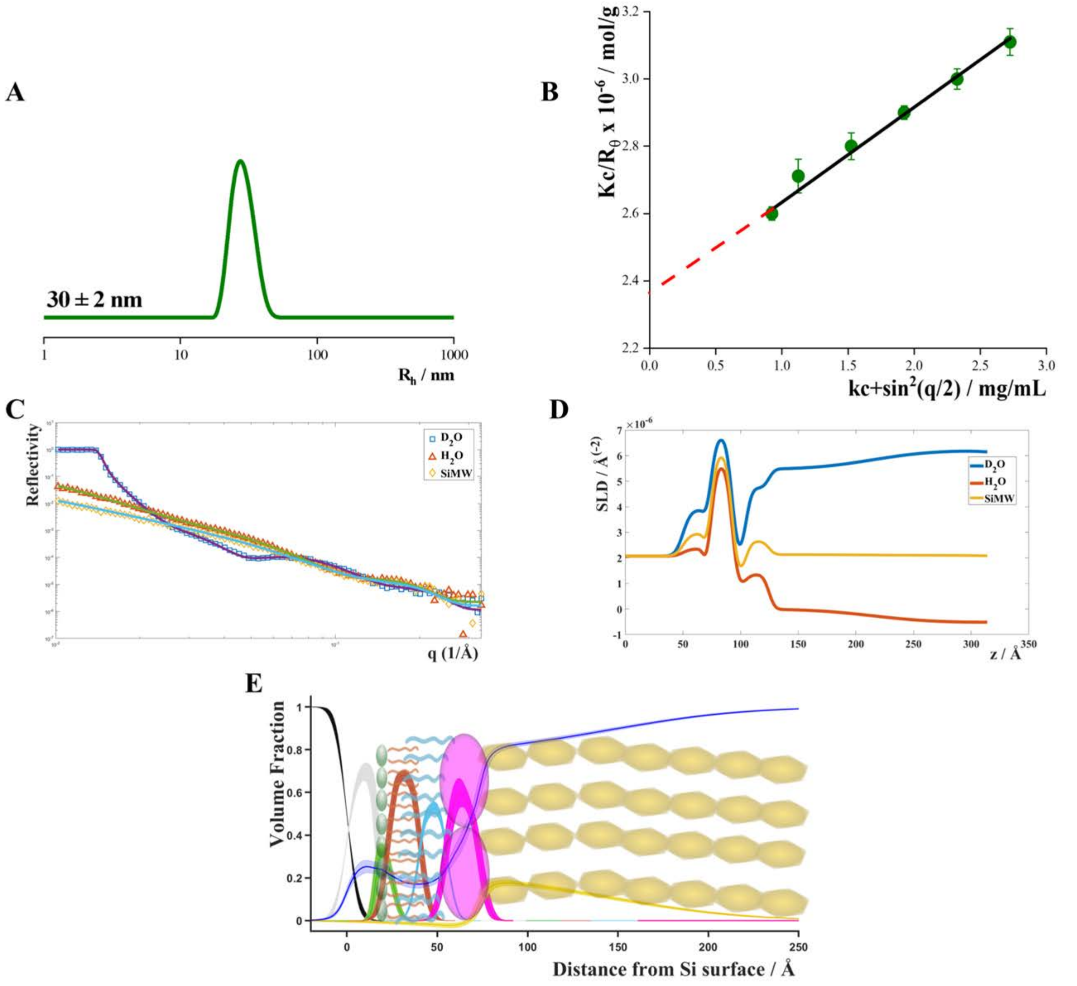

A
30 ± 2 nm
R_h / nm
B
Kc/R_θ x 10^-6 / mol/g
kc+sin^2(q/2) / mg/mL
C
Reflectivity
D_2O
H_2O
SiMW
q (1/Å)
D
SLD / Å^(-2)
D_2O
H_2O
SiMW
z / Å
E
Volume Fraction
Distance from Si surface / Å
